# Species-level virome profiling reveals compositional differences in the gut prokaryotic DNA virome of people with HIV-1 on antiretroviral therapy

**DOI:** 10.1080/29933935.2026.2688064

**Published:** 2026-06-18

**Authors:** Ji Zhang, Andreas Matussek, Anders Sönnerborg, Xiangning Bai

**Affiliations:** a Fonterra Research and Development Centre, Palmerston North, New Zealand; b Department of Medicine Huddinge, Centre for Infectious Medicine, Karolinska Institutet, Huddinge, Sweden; c Department of Microbiology, Division of Laboratory Medicine, Oslo University Hospital, Oslo, Norway; d Department of Microbiology, Division of Laboratory Medicine, Institute of Clinical Medicine, University of Oslo, Oslo, Norway; e Department of Clinical Microbiology, Karolinska University Hospital, Stockholm, Sweden

**Keywords:** Gut virome, prokaryotic DNA viruses, bacteriophages, HIV-1, antiretroviral therapy

## Abstract

Alterations in the gut bacterial communities are well described in people with HIV-1 (PWH). However, less is known about the gut viral communities (virome). Herein, we performed an exploratory study of the gut DNA virome in PWH receiving antiretroviral therapy (PWH-ART). Virome profiling was performed using two complementary viral taxa identification pipelines, MetaPhlAn4 and Phanta, providing high-resolution taxonomic profiles at the species level. Consistent patterns of the gut DNA virome were obtained with both pipelines, though Phanta identified a greater number of viral taxa at the species level. Most gut DNA viruses were unclassified species, and bacteriophages constituted the majority of identifiable viral populations. Compared to HIV-1 negative controls (*n* = 11), PWH‑ART (*n* = 13) exhibited compositional differences in their gut DNA virome at finer taxonomic levels (species and genus), including higher within-group variations and multiple differentially abundant viral taxa. Gender influenced the virome composition, independent of HIV-1 status and age. Furthermore, correlations were identified between multiple unclassified viral species and clinical variables, including gender, CD4+ T cell counts, and ART duration. Further studies are warranted to validate these findings and to elucidate whether the observed compositional differences in the gut DNA virome are mediated by HIV-1 infection itself, by ART, or by both.

## Introduction

The human gut contains diverse microorganisms comprised of viruses, bacteria, archaea, and fungi. Studies, including ours, have so far focused primarily on the gut bacterial community (bacteriome) and its effects on human health and disease, including in people with immunodeficiency virus type 1 (HIV-1) infection (PWH),^1-3^ while other microbial components, such as viral communities (virome), have been largely neglected.^4^ The human gut virome is a complex ecosystem consisting of viruses infecting prokaryotic and eukaryotic cells, which are commonly termed as prokaryotic and eukaryotic viruses.^5^ ^,^ ^6^ Despite the rapid development of culture-independent techniques, which has remarkably advanced our understanding of the human gut microbiome, the research on the gut virome is in its early days.^4^ The gut prokaryotic viruses (predominantly bacteriophages), which constitute >90% of the gut virome, are poorly characterized, largely owing to methodological limitations in successfully isolating bacteriophages and genome annotations.^7-9^ Consequently, a vast majority of the viral sequences share little to no homology with reference databases.^9^ In addition, most bioinformatics and analytical tools in the microbiome field were originally designed for analyzing the bacteriome (the main component of the microbiome), while no standard methods are presently available for virome characterization. As such, cataloging viruses is merely the beginning of uncovering the gut virome.

Recent advancements in the knowledge of the virome have been achieved by a multitude of emerging computational tools and reference databases, as well as the practical procedures of cataloging the virome based on thousands of metagenome datasets.^10^ ^,^ ^11^ These methodological advancements collectively provide compelling evidence that the gut virome plays critical roles in human health by shaping microbial community, modulating host immune responses, and influencing metabolic pathways.^5^ Differences in the gut virome are increasingly being associated with e.g., chronic immune and inflammatory conditions, metabolic disorders, neurological conditions and colorectal cancer.^5^ ^,^ ^12-15^ In the context of HIV-1 infection, a few studies have demonstrated associations between HIV-1 infection, immunodeficiency, and alterations in the gut virome, in particular, the eukaryotic virome.^16-18^ Specifically, a profound expansion in several human eukaryotic viruses, such as Anelloviridae, Adenoviridae, and Papillomaviridae, has been reported to be associated with HIV-1 infection.^16-18^ However, the prokaryotic virome, predominantly bacteriophage communities, remain largely unchanged according to these studies, of which one study only reported a decrease in Inoviridae bacteriophage sequences, but no major change in overall bacteriophage populations.^16^ The lack of consistent and significant findings in the prokaryotic virome likely stems from the fundamental methodological challenges in bacteriophage characterization, such as the lack of standardized protocols and comprehensive reference databases as noted above.^7-9^ It is noteworthy that methodological choices in viral detection critically influence results and biological interpretation of the gut virome in health and disease.^5^ Moreover, current virome studies, including a few HIV-related ones, are often limited to viral taxonomies at the family level. Given the extraordinarily diverse and pervasively mosaic nature of bacteriophages, their functional properties can vary significantly even among closely related species or strain variants.^19^ Therefore, virome profiling at finer taxonomic resolution is crucial to elucidate a precise gut virome landscape in relation to HIV-1 infection and other health conditions.

The aim of our study was to perform an exploratory characterization of the gut DNA virome in people with HIV (PWH), compared to HIV-1 negative healthy controls, with a particular focus on the dominant prokaryotic DNA virome. To achieve this goal, we evaluated the usefulness of two complementary pipelines for virome profiling: the marker gene-based MetaPhlAn4,^18^ ^,^ ^19^ and the phage-inclusive gut microbiome profiler Phanta.^20^ Both pipelines offer high-resolution taxonomic profiles down to the species level, using different profiling strategies. It should be noted that all PWH in this exploratory cohort were on antiretroviral therapy (ART) at the time of fecal sampling; thus, our study was not aimed at establishing associations between the gut DNA virome and HIV-1 infection alone, but rather to characterize the gut DNA virome in relation to HIV-1 infection and ongoing ART, using a cohort of PWH on ART (PWH-ART), as compared to HIV-1 negative controls. The obtained data are expected to provide references for future comprehensive studies investigating the gut virome and its role in HIV-1 infection, ultimately paving the way toward potential novel therapeutic strategies.

## Materials and methods

### Study subjects and sample collection

This study included 13 PWH-ART (median: 45 y; range: 38-62 y) who were recruited from the out-patient clinic at Karolinska University Hospital, Stockholm, Sweden, and 11 HIV-1 negative healthy individuals as controls (median: 32 y; range: 24–51 y) who did not receive antibiotic treatment during the last 3 months, as described.^3^ The PWH-ART participants had been given ART for a median of 7.7 y with combinations of abacavir/lamivudine/dolutegravir (*n* = 4), tenofovir disoproxil fumarate (TDF)/emtricitabine/dolutegravir (*n* = 3), TDF/emtricitabine/rilpivirine (*n* = 2), tenofovir/emtricitabine/rilpivirine (*n* = 1), tenofovir/emtricitabine/dolutegravir (*n* = 1), TDF/emtricitabine/efavirenz (*n* = 1), or dolutegravir/abacavir/lamivudine (*n* = 1).^3^ None of them had ongoing HIV-1-related complications, antibiotic treatment, or gastrointestinal complaints during the previous 3 months before fecal sampling. Fecal samples and clinical data of PWH-ART, including gender, age, current and nadir CD4+ T cell counts, and ART duration were collected as described previously.^3^ The current CD4+ T cell count was measured at the time of fecal sampling, and the nadir CD4+ T cell count was defined as the lowest CD4+ T cell count ever registered for a patient, which was measured at diagnosis and before initiation of ART. The study was approved by the Regional Ethics Committee, Stockholm (2009/1485-31, 111 2013/1944-31/4, 2014/920-3). All participants provided informed consent to participate in this study.

### Metagenomic data pre-processing

Sample preparation and DNA shotgun metagenome sequencing were performed as described previously.^3^ The raw sequencing data were previously submitted to the NCBI SRA database with BioProject ID PRJNA692830,^3^ and were downloaded for the present study. The raw sequencing reads were processed as described previously.^3^ In brief, trim_galore (version: 0.6.10) was used for adapter trimming and removal of low-quality reads. The processed reads were mapped to the human genome database using KneadData (version 0.1; https://github.com/biobakery/kneaddata) to identify and remove human-derived sequences. This step may also remove reads derived from integrated viral sequences that are present in the human reference genome. Therefore, while eukaryotic viruses (e.g., from other hosts) may be detected, the core analyses and interpretations of this study focus on the less studied but dominant prokaryotic DNA virome.

### DNA virome profiling using two bioinformatics pipelines

Two bioinformatics pipelines, MetaPhlAn (v4.1.1)^20^ ^,^ ^21^ and Phanta (v1.1.1),^22^ were used to identify and quantify sequences of the microbial communities. MetaPhlAn is a widely used metagenomics tool that is capable of comprehensively profiling viruses, bacteria, archaea, and eukaryotes. It uses a marker gene-based approach, and since its recent version 4.1, it has extended its marker-based paradigm to capture viral diversity in the human gut microbiome.^20^ ^,^ ^21^ In our study, the database version mpa_vJun23_CHOCOPhlAnSGB_202403 was used for microbial community profiling. A minimum 75% breadth-of-coverage for its marker genes to be considered as “detected” is required in the analysis, and viral sequences with a breadth-of-coverage <75% were excluded by the pipeline. Viral sequence groups (VSGs), which are analogous to species-level operational clusters, are classified via a graph clustering approach, as described previously.^20^ ^,^ ^21^ The taxonomy output from MetaPhlAn4 is non-hierarchical, because it utilizes unique clade-specific marker genes to identify taxa, and hierarchical relationships between taxa are not required in its classification; therefore, only the VSGs level would be assigned by MetaPhlAn4 and included in subsequent analyses.

In comparison, Phanta uses a k-mer-based approach and has been optimized for detailed virome analysis, particularly for gut viruses.^22^ We used the Phanta default settings and database, which is reported to be best suited for the analysis of fecal metagenomes. Phanta assigns species-level viral operational taxonomic units (vOTUs). vOTUs with high similarity to a known viral species were labeled with the NCBI-assigned taxonomy of that species. In our analysis, Phanta considered a viral species as “true positive” if the genome coverage was above 10%, and viral sequences with low genome coverage (<10%) were excluded. Unlike MetaPhlAn4, Phanta is built on Kraken2, whose classification is inherently hierarchical as it relies on a taxonomic tree structure to map k-mers to their lowest common ancestor. Therefore, higher taxonomic levels of each vOTU were assigned iteratively by Phanta, as described.^22^

### Downstream and statistical analysis

Baseline characteristics were compared, using the Wilcoxon rank-sum test for continuous variables (age, nadir and current CD4+ T-cell counts, ART duration), and Fisher's exact test for categorical variable gender (male versus female). PWH-ART presented with statistically older age (median: 45 y) than controls (median: 32 y) (Wilcoxon rank sum test: *p* < 0.001) and were overrepresented by females (69%), while all controls were males (Fisher’s exact test: *p* < 0.001).

We first performed single-factor analyses between outcome groups: PWH-ART versus controls. Within-sample diversity (alpha diversity) was measured using the R function *vegan:diversity*. All alpha diversity indices were calculated, and two indices representing different aspects of alpha diversity were presented: the Shannon index (richness and evenness) and InvSimpson index (dominance). The Wilcoxon rank-sum test was used to test the statistical significance (*p-*value) of group differences. Beta diversity (overall virome composition) was measured by Bray‒Curtis distances using R package Phyloseq (1.55.2). Permutational multivariate analysis of variance (PERMANOVA) was performed to test the differences in the virome composition using the *vegan:adonis2* function with 9999 permutations, based on Bray–Curtis distances. Effect size (R^2^: proportion of variance in the gut virome composition explained by one variable) and statistical significance were obtained by PERMANOVA. A principal coordinate analysis (PCoA) plot was used to assess the distribution of the data in two dimensions based on Bray–Curtis distances. To assess whether any significant PERMANOVA results could be attributed to differences in within-group dispersion rather than centroid (average composition) shifts, we additionally performed permutational analysis of multivariate dispersions (PERMDISP) based on Bray–Curtis distances, and statistical significance was determined using permutation tests with 9999 permutations. Differences in the diversity matrix with *p-*value < 0.05 were considered statistically significant, while differences with values of 0.05 < *p* < 0.1 indicated a statistical trend. Differential abundance analysis was performed using ANCOM-BC (2.13.1).^23^ Viral taxa with *p* < 0.05 were considered statistically significantly different between groups. To assess correlations between viral taxa identified by the two pipelines and clinical variables, we adopted a stratified analytical approach, based on sample size and variable type. Correlations between continuous clinical variables (age, CD4+ T‑cell counts, and ART duration) and viral taxa were assessed using Spearman’s rank correlation. For variables available for all participants (age), analysis was performed on the full cohort (*n* = 24). For variables available only for PWH‑ART (CD4+ T‑cell counts, ART duration), the analysis was restricted to the PWH‑ART subgroup (*n* = 13). Association between categorical variable gender and viral taxa was analyzed using the Wilcoxon rank‑sum test, a non‑parametric method assessing whether viral abundance differs between independent groups (males and females). This analysis included all participants (*n* = 24). To avoid sparse taxa that could generate unstable correlation estimates, viral taxa with a prevalence >10% across all samples were included in the analyses. Spearman’s correlation coefficients and Wilcoxon rank‑sum test with *p* < 0.05 were considered statistically significant.

Given the statistical differences in age and gender between PWH-ART and controls, we further performed analyses adjusting for the covariates to establish the independent associations of the gut virome with outcome status/group (PWH-ART versus controls), as well as with covariates (i.e., age and gender). Specifically, for alpha diversity, analysis of covariance (ANCOVA) was performed with the model “diversity ~ outcome group + age + gender” using type II sums of squares (car:Anova) to assess the independent effect of each variable on the gut virome after adjusting for the others. For beta diversity, PERMANOVA was performed using the model “bray_dist ~ outcome group + age + gender” with the by = “margin” parameter to test the unique contribution of each variable after adjusting for the others. For differential abundance analysis, ANCOM-BC with the formula = “outcome group + age + gender” was used to test the differences between outcome groups, as well as between genders, after adjusting for other variables. For correlations between age and the relative abundance of viral taxa among all participants (*n* = 24), partial Spearman correlation analysis adjusted for gender was performed. For correlations between gender and viral taxa among all participants, linear regression‑based partial correlation (equivalent to a point‑biserial partial correlation) adjusted for age was performed. Correlations between viral taxa and clinical variables available only among PWH-ART participants (CD4+ T-cell counts, ART duration) were performed using Spearman correlations without covariate adjustment, due to the limited sample size of the PWH-ART group (*n* = 13). Multiple testing corrections were not applied because of the small sample size and the exploratory focus of the study.

## Results

### Gut DNA virome is dominated by unknown viruses and bacteriophages constitute the majority of the identifiable viral populations

**Figure 1.**
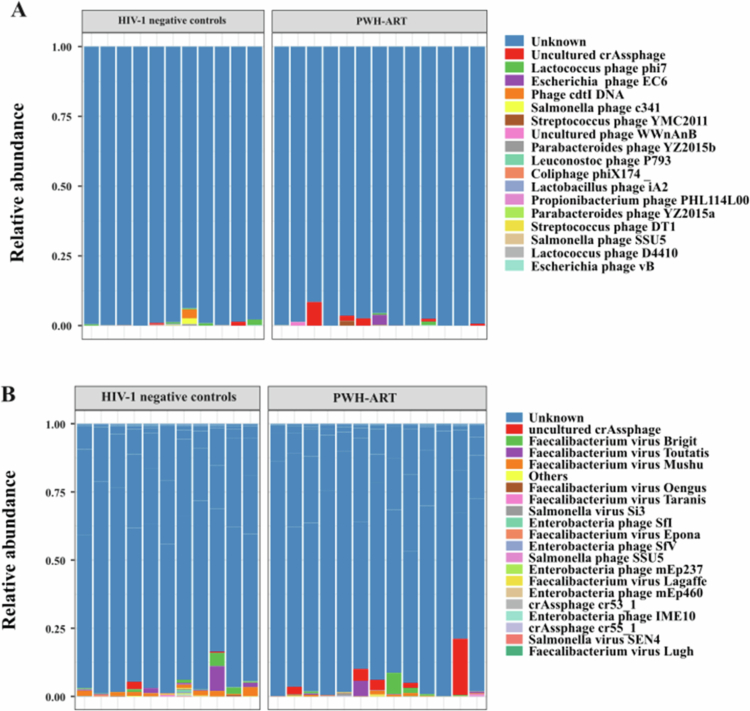
Viral composition identified in 13 PWH-ART and 11 HIV-1 negative controls. (A) Composition and relative abundance of all Viral Sequence Groups (VSGs) identified using the MetaPhlAn4 pipeline. Seventeen VSGs of known viruses are indicated. “Unknown” corresponds to 274 VSGs of unknown viruses. Taxa are ordered by their decreasing total relative abundance across samples. A clearer visualization of 17 VSGs of known viral species is presented in the supplementary material Figure S1A. (B) Composition and relative abundance of all species-level viral operational taxonomic units (vOTUs) identified using the Phanta pipeline with its default database. Taxa are ordered by their decreasing total relative abundance across samples. The top 19 of 43 vOTUs assigned to known viral species are indicated. “Others” corresponds to the remaining 24 vOTUs of known viral species. “Unknown” corresponds to 4232 vOTUs of unknown species. A clearer visualization of 43 vOTUs of known viral species is presented in the supplementary Figure S1B.

In our 24 fecal samples, 291 Viral Sequence Groups (VSGs) were identified using the MetaPhlAn4 pipeline. Of these, 274 (94%) VSGs were unmapped to any known viral genomes, and only 17 (6%) VSGs were assigned to known viral genomes; all belonged to bacteriophages (Figure 1A and Supplementary Figure S1A). Using the Phanta pipeline with its default database, 4275 unique species-level viral operational taxonomic units (vOTUs) were identified. Of these, only 43 vOTU (1%) were assigned to known viral species, and all were bacteriophages (Figure 1B and Supplementary Figure S1B).

At taxonomic levels above the species assigned using the Phanta pipeline (supplementary Figure S2), Brigitvirus, Toutatisvirus, Mushuvirus, Lilyvirus, and Svunavirus were the top five identifiable known genera, followed by the other viral genera shown in the supplementary Figure S2. Siphoviridae, Myoviridae, Podoviridae, Inoviridae, Microviridae, Autographiviridae, Drexlerviridae, and Circoviridae are identifiable viral families. Caudovirales was the most abundant identifiable viral order, followed by Tubulavirales, Petitvirales, and Cirlivirales. Caudoviricetes was the most abundant identifiable viral class, followed by Faserviricetes, Malgrandaviricetes, and Arfiviricetes. Uroviricota was the most abundant identifiable viral phylum, followed by Hofneiviricota, Phixviricota, and Cressdnaviricota. Only one eukaryotic viral taxon, i.e., an unknown species within the Circovirus genus, Circoviridae family, Cirlivirales order, Arfiviricetes class, and Cressdnaviricota phylum, was identified from one PWH participant.

### Gut DNA virome diversity differs in PWH-ART and associates with gender

**Figure 2.**
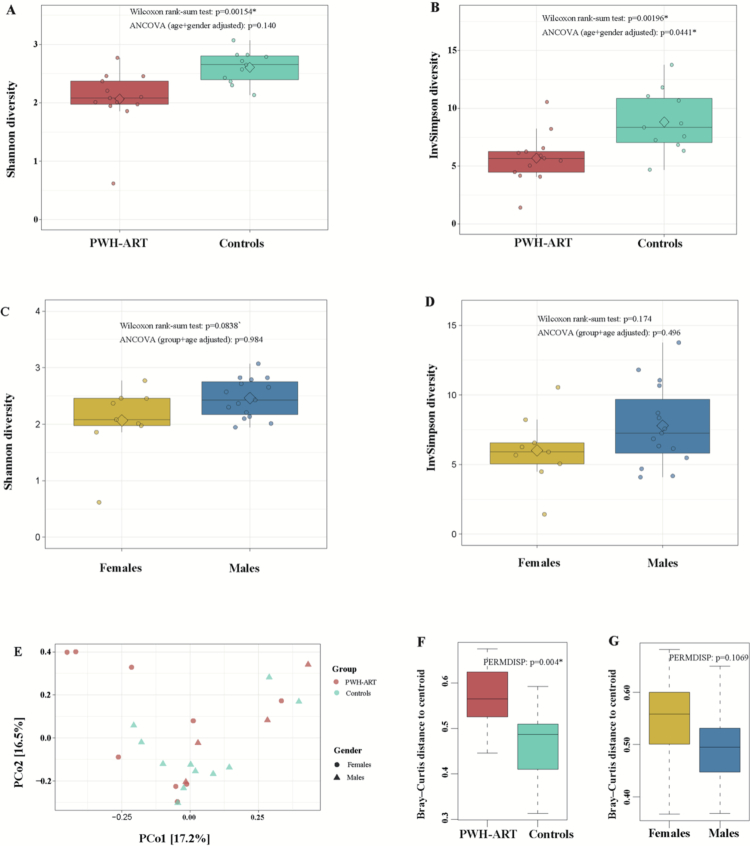
Difference in gut DNA virome diversity at species-level Viral Sequence Groups (VSGs) characterized using the MetaPhlAn4 pipeline, between outcome groups (PWH-ART and controls) and genders (males and females). (A, B) Shannon and InvSimpson diversity between PWH-ART and controls. (C, D) Shannon and InvSimpson diversity between males and females. Boxplots display the median (center line), mean (diamond), interquartile range (box bounds), and individual values (dots). *p*-values were calculated using Wilcoxon rank-sum test without covariates adjustment, and analysis of covariance (ANCOVA) adjusted for covariates. (E) Beta diversity illustrated by principal coordinates analysis (PCoA) based on Bray‒Curtis distances. Points are coloured by outcome group and gender, as shown. The principal coordinate (PCo) values (PCo1 on the x-axis and PCo2 on the y-axis) represent the percent of total variation explained by that axis. Permutational analysis of multivariate dispersions (PERMDISP) of within-group dispersion based on Bray–Curtis distances, between PWH-ART and controls (F), and between males and females (G). Boxplots show distances of individual samples to their respective group centroid. *p-*values were calculated using permutation tests (9999 permutations). *Statistically significant difference (*p* < 0.05), ´difference with a statistical trend (0.05 < *p* < 0.1).

Based on the virome profile identified using MetaPhlAn4, a statistically lower Shannon diversity (Wilcoxon rank-sum test: *p* = 0.00154) and InvSimpson diversity (Wilcoxon rank-sum test: *p* = 0.00196) were observed in PWH-ART compared to HIV-1 negative controls before covariate adjustment. After adjusting for age and gender, InvSimpson diversity remained statistically lower in PWH-ART (ANCOVA: *p* = 0.044), while Shannon diversity lost statistical difference between outcome groups (ANCOVA: *p* = 0.14) (Figure 2A, B). Alpha diversity appeared to be higher in males compared to females; the difference was not statistically significant after adjusting for outcome group and age (Figure 2C, D).

Beta diversity (virome composition), assessed with Bray–Curtis distances, significantly differed between PWH-ART and that in HIV-1 negative controls before covariate adjustment (PERMANOVA: R^2^ = 0.0719, *p* = 0.0379) (Table 1). After adjusting for age and gender, the difference in virome composition showed a statistical trend between PWH-ART and controls (PERMANOVA: R^2^ = 0.064, *p* = 0.0605) (Table 1). Notably, virome composition significantly differed between males and females after adjusting for outcome group and age (PERMANOVA: R^2^ = 0.073, *p* = 0.0324), while age showed no effect on virome composition after adjusting for outcome group and gender (PERMANOVA: R^2^ = 0.0318, *p* = 0.733) (Table 1). PCoA showed that the virome composition from PWH-ART and females was more diversely distributed compared to that from HIV-1 negative controls and males, respectively (Figure 2E). Within-group dispersion was statistically higher in PWH-ART compared to controls (PERMDISP: *p* = 0.004) (Figure 2F), suggesting that within-group dispersion may contribute partially to the significant PERMANOVA result between PWH-ART and controls. No statistical difference in within-group dispersion was observed between males and females (PERMDISP: *p* = 0.1069) (Figure 2G), indicating that the significant PERMANOVA result between genders is not driven by within-group dispersion, but reflects the genuine differences in centroid (average composition) between males and females.

**Figure 3.**
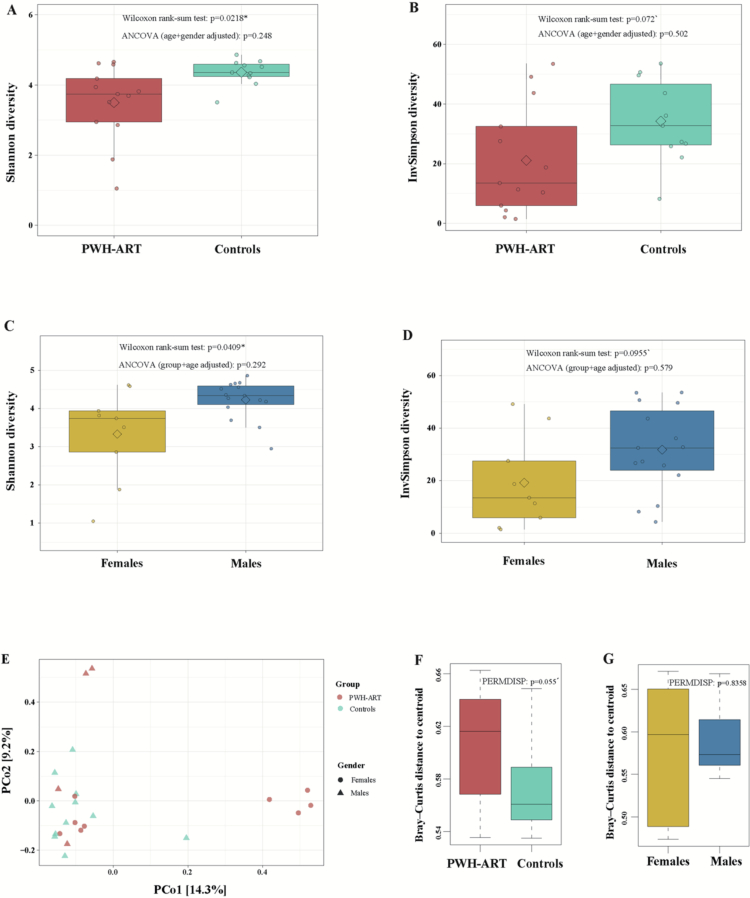
Difference in gut DNA virome diversity at the species-level viral operational taxonomic units (vOTUs) characterized using the Phanta pipeline between outcome groups (PWH-ART and controls) and genders (males and females). (A, B) Shannon and InvSimpson diversity between PWH-ART and controls. (C, D) Shannon and InvSimpson diversity between males and females. Boxplots display the median (center line), mean (diamond), interquartile range (box bounds), and individual values (dots). *p*-values were calculated using Wilcoxon rank-sum test without covariates adjustment, and analysis of covariance (ANCOVA) adjusted for age and gender. E, Beta diversity illustrated by principal coordinate analysis (PCoA) based on Bray‒Curtis distances. Points are coloured by group and gender, as shown. The principal coordinate (PCo) values (PCo1 on the x-axis and PCo2 on the y-axis) represent the percent of total variation explained by that axis. Permutational analysis of multivariate dispersions (PERMDISP) of within-group dispersion based on Bray‒Curtis distances between PWH-ART and controls (F) and between males and females (G). Boxplots show distances of individual samples to their respective group centroid. *p-*values were calculated using permutation tests (9999 permutations). *Statistically significant difference (*p* < 0.05), ´difference with a statistical trend (0.05 < *p* < 0.1).

Similar results were observed from analyses based on the virome profile identified using the Phanta pipeline. Specifically, PWH-ART presented a lower alpha diversity of species-level vOTUs before covariate adjustments. After adjusting for age and gender, the group differences in alpha diversity lost statistical significance (Figure 3A, B). Males presented higher Shannon and InvSimpson diversity compared to females before covariates adjustment (Wilcoxon rank-sum test: *p* = 0.0409 and 0.0955), and after adjusting for outcome group and age, the difference lost statistical significance (ANCOVA: *p* > 0.2) (Figure 3C, D). However, virome composition differed significantly between PWH-ART and controls, both before and after adjusting for age and gender (PERMANOVA: *p* < 0.05) (Table 1). Gender significantly influenced virome composition, both before and after adjusting for outcome group and age, while age had no effect on virome composition (Table 1). PCoA showed that the virome composition from PWH-ART and females was more diversely distributed compared to that from controls and males, respectively (Figure 3E). PERMDISP further showed that the within-group dispersion appeared to be higher in PWH-ART than in controls, with a statistical trend (PERMDISP: *p* = 0.055) (Figure 3F), indicating that the within-group variance may contribute partially to the significant PERMANOVA result between PWH-ART and controls. However, no statistical difference in within-group dispersion was found between males and females (PERMDISP: *p* = 0.8358) (Figure 3G), supporting that the compositional differences between genders represent the genuine differences in their average compositions, rather than within-group variations.

Taken together, the consistent patterns of virome diversity based on virome profiles with both pipelines suggest that age and gender explain much of the variations in within‑sample virome diversity (alpha diversity); however, the overall gut virome composition (beta diversity) is independently associated with PWH-ART status (or HIV-1 infection and ART), and gender independently influences gut virome composition.

**Table 1.** Single-factor and covariate-adjusted PERMANOVA analysis.

MetaPhlAn4	Single-factor		Covariates-adjusted^#^	
	R^2^ value	*p-*value	R^2^ value	*p-*value
Outcome group	0.07186	0.0379*	0.06402	0.0605**`**
Gender	0.09479	0.0025*	0.07268	0.0324*
Age	0.04568	0.3813	0.0318	0.733
**Phanta**				
Outcome group	0.06249	0.0339*	0.0617	0.0201*
Gender	0.07298	0.0045*	0.0661	0.0109*
Age	0.04774	0.23	0.0407	0.4571

^#^ The effect of each variable was adjusted for the others: outcome group adjusted for gender and age; age adjusted for outcome group and gender; gender adjusted for outcome group and age.

*Statistically significant difference (*p* < 0.05).

**´**Difference with a statistical trend (0.05 < *p* < 0.1).

We further assessed viral alpha and beta diversity across taxonomic levels above species (genus, family, order, class, and phylum) assigned using the Phanta pipeline. Similarly, no statistical difference in alpha diversity indices was observed between groups at other taxonomic levels after adjusting for age and gender (ANCOVA: *p* > 0.1 for all indices). Genus-level virome composition assessed with Bray‒Curtis distances was statistically different between PWH-ART and controls, after adjusting for age and gender (PERMANOVA: R^2^ = 0.0662, *p* = 0.0468), while not at other taxonomic levels (PERMANOVA: *p* > 0.05). Gender significantly influenced the gut virome composition at the genus level, after adjusting for outcome group and age (PERMANOVA: R^2^ = 0.0803, *p* = 0.0153), but not at other taxonomic levels (family to phylum) (PERMANOVA: *p* > 0.05). Age did not influence virome composition at any taxonomic level (PERMANOVA: *p* > 0.05). These results demonstrate that the gut virome composition differs significantly at finer taxonomic levels (i.e., species and genus), but not at broader levels (family to phylum), between PWH-ART and controls, and that gender independently influences the gut virome composition at these finer taxonomic levels.

### Differentially abundant viruses between outcome groups and genders

**Figure 4.**
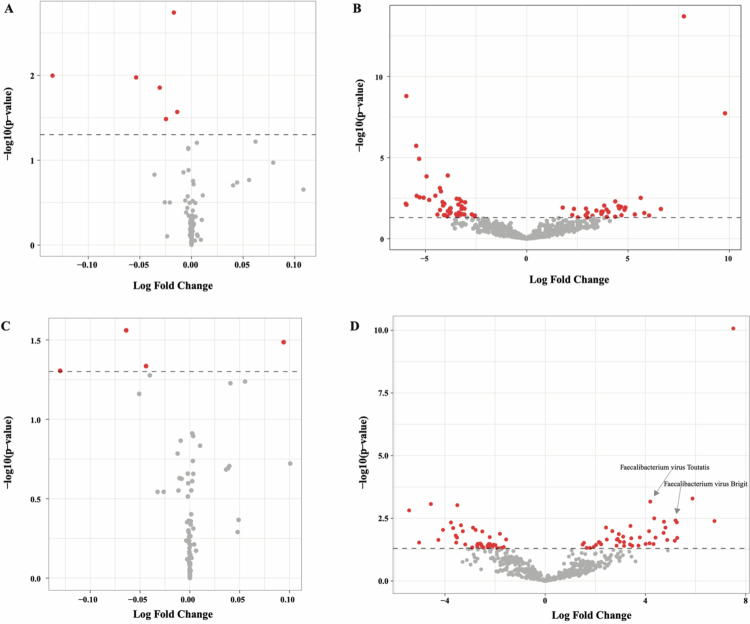
Differentially abundant viral taxa between outcome groups (A, B) and genders (C, D). Analyses were performed using ANCOM-BC, adjusted for covariates (outcome group adjusted for age and gender; gender adjusted for outcome group and age). Volcano plots showing (A) differences in 291 species-level Viral Sequence Groups (VSGs) identified by MetaPhlAn4 pipeline between PWH-ART and controls, adjusted for age and gender; (B) differences in 4257 species-level viral operational taxonomic units (vOTUs) identified by Phanta pipeline between PWH-ART and controls, adjusted for age and gender; (C) differences in 291 species-level VSGs identified by MetaPhlAn4 pipeline between males and females, adjusted for outcome group and age; (D) differences in 4257 species-level vOTUs identified by Phanta pipeline between males and females, adjusted for outcome group and age.Log Fold Change (LFC) is shown on the x-axis and log10 of *p*-value is shown on the y-axis. Red dots indicate statistically differentially abundant VSGs/vOTUs between groups (*p* < 0.05). A, B: dots with LFC > 0 represent VSGs/vOTUs that were more abundant in PWH-ART, while dots with LFC < 0 represent VSGs/vOTUs that were less abundant in PWH-ART. C, D: dots with LFC> 0 represent VSGs/vOTUs that were more abundant in males, while dots with LFC< 0 represent VSGs/vOTUs that were less abundant in males. Known viral species are indicated as shown, while all other differentially abundant VSGs/vOTUs (red dots) are unknown species. Taxonomic details of each differentially abundant VSG/vOTU are presented in the supplementary Table S1.

Among the 291 VSGs identified using the MetaPhlAn4 pipeline, three VSGs of unknown species (M229, M756, and M892) showed statistically lower relative abundance in PWH-ART compared to controls before covariates adjustment. After adjusting for age and gender, six VSGs of unknown species, namely, M697, M892, M460, M756, M459, and M914, showed statistically lower relative abundance in PWH-ART compared to controls, with low fold changes (log fold change < –0.15, *p* < 0.05) (Figure 4A and supplementary Table S1A). Among the 4275 vOTUs identified using the Phanta pipeline with its default database, 82 vOTUs were differentially abundant between PWH-ART and controls before covariates adjustment. After adjusting for age and gender, 79 vOTUs were differentially abundant between outcome groups, among which 34 were more abundant in PWH-ART, while 45 vOTUs were less abundant in PWH-ART compared to controls (Figure 4B and supplementary Table S1B). All these 79 vOTUs were unknown viral species within the *Caudovirales* order, except one unknown order (supplementary Table S1B).

Given that gender independently influenced the gut virome composition in this cohort, analysis was further performed between genders. Four VSGs of unknown species identified by the MetaPhlAn4 pipeline (Figure 4C and supplementary Table S1C) and 88 vOTU (86 of unknown species and two of known species) identified by the Phanta pipeline (Figure 4D and supplementary Table S1D) were differentially abundant between males and females. The two known viral species, i.e., Faecalibacterium virus Toutatis and Faecalibacterium virus Brigit identified by the Phanta pipeline were more abundant in males than in females (log fold change > 4, *p* < 0.005) (Figure 4D and supplementary Table S1D).

We additionally performed analysis on taxonomic levels above species assigned by the Phanta pipeline between PWH-ART and controls, adjusted for age and gender. At the genus level, 63 differentially abundant genera were identified between PWH-ART and controls, among which one known genus, Lubbockvirus (log fold change = 2.68, *p* = 0.0465), and 37 unknown genera were more abundant, while 25 unknown genera were less abundant in PWH-ART compared to controls. At the family level, Inoviridae was less abundant in PWH-ART (log fold change = −2.99, *p* = 0.01). At the order level, Petitvirales (log fold change = −2.49, *p* = 0.047) and Tubulavirales were less abundant in PWH-ART (log fold change = -3.10, *p* = 0.0025). At the class level, Malgrandaviricetes (log fold change = -2.49, *p* = 0.047) and Faserviricetes were less abundant in PWH-ART (log fold change = −3.10, *p* = 0.0025). At the phylum level, Phixviricota (log fold change = −2.49, *p* = 0.047) and Hofneiviricota were less abundant in PWH-ART (log fold change = −3.10, *p* = 0.0025).

### Correlations between viral taxa and clinical variables

**Figure 5.**
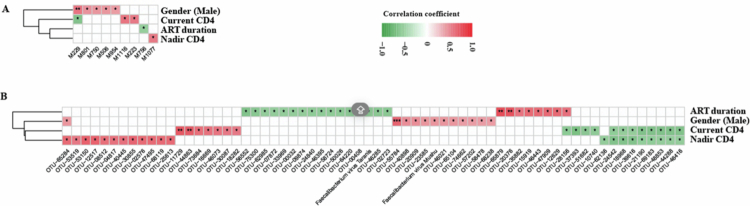
Correlations between clinical variables and viral taxa at species-level Viral Sequence Groups (VSGs) with prevalence >10% identified using MetaPhlAn4 (A), and at species-level viral operational taxonomic units (vOTUs) with prevalence >10% identified using Phanta (B). For continuous clinical variables (age, nadir, and current CD4+ T‑cell count, ART duration), the color gradient represents Spearman’s rank correlation coefficient. For the categorical variable gender, the color gradient represents the age‑adjusted partial correlation coefficient. Both correlation coefficients range from −1 to +1, with red indicating a positive correlation (higher abundance in males for gender) and green indicating a negative correlation (higher abundance in females for gender). Statistically significant correlations are indicated with asterisk: *p < 0.05 and **p < 0.01. Clinical variables with similar correlation patterns are clustered by *pheatmap()* using hierarchical clustering.

We assessed any correlations between species-level viral taxa with prevalence > 10% (i.e., present in at least 10% of all samples) and clinical variables. Among the 81 VSGs with prevalence > 10% identified using the MetaPhlAn4 pipeline, 13 VSGs correlated to one or more clinical variables before covariate adjustment. After covariate adjustment, correlations between age and viral taxa disappeared, while gender (male) was positively correlated to five VSGs (Figure 5A). Among PWH-ART participants, the current CD4+ T-cell count was positively correlated to two VSGs, and inversely correlated to one VSG; nadir CD4+ T-cell count was positively correlated to one VSG; and the ART duration was inversely correlated to one VSG (Figure 5A).

Among the 661 vOTUs with prevalence >10% identified using the Phanta pipeline, correlations were observed between multiple vOTUs and clinical variables. Consistent with findings based on viral taxa from MetaPhlAn4 above, correlations between age and viral taxa disappeared after adjusting for gender, while gender (male) remained positively correlated to several viral taxa after adjusting for age (Figure 5B). Among PWH-ART participants, ART duration was positively correlated to eight vOTUs and inversely correlated to 16 vOTUs; CD4+ T-cell counts correlated to several vOTUs, eight of them correlated inversely to both nadir and current CD4+ T-cell counts (Figure 5B).

## Discussion

Our study confirms that most gut DNA viruses are unclassified viral species, and that bacteriophages constitute the majority of the identifiable viral populations, with most belonging to the order Caudovirales (mainly the Siphoviridae, Myoviridae, and Podoviridae families), aligning with prior reports both among PWH and the general population.^17^ ^,^ ^24^ ^,^ ^25^ Notably, only one eukaryotic viral taxon – an unclassified species within the Circovirus genus (family Circoviridae), was identified from one PWH participant. The scarcity of identifiable eukaryotic viruses in our results may be attributed to several factors. One is a technical consequence of the bioinformatic filtering applied: sequences from eukaryotic viruses that share high similarity with the human genome or are integrated into it are removed during the standard host-read depletion step. Additionally, positive detections may exhibit low genome coverage (Phanta) or limited breadth of coverage (MetaPhlAn4), leading to their subsequent exclusion by the pipelines. Therefore, while the presence of Circovirus is noted, the core analyses and interpretations of our study focus on the prokaryotic DNA virome – dominant yet poorly characterized viral component, as compared to eukaryotic viruses, which are known to represent a tiny fraction of the gut virome.^7^ ^,^ ^26^

Our study demonstrates significant compositional differences in the gut DNA virome in PWH-ART compared to HIV-1 negative controls, characterized by statistical differences in beta diversity (overall virome composition) and differential abundance of multiple viral taxa of unknown species. This pattern remains after adjusting for the significant covariates of age and gender. In addition, PWH-ART showed higher variations in their gut DNA virome composition (increased community dispersion/beta diversity) compared to uninfected controls, aligning with our previous findings in the gut bacteriome in the same cohort,^27^ and also with other reports where HIV-1 infection and ART were found to be associated with increased beta diversity of the gut bacteriome.^28^ These data suggest that HIV-infection and/or ART drive the shift toward a more dissimilar or ´dysbiotic´ gut microbiome structure. Unlike beta diversity, alpha diversity between PWH-ART and controls lost statistical significance after adjusting for age and gender. This suggests that host age and gender are important confounders influencing within-sample diversity, but they do not account for the compositional differences between PWH-ART and controls in our cohort, supporting that PWH-ART status (i.e., HIV‑1 infection and ART) drives shifts in the gut DNA virome composition, independently of gender and age.

Multiple viral taxa were found to be differentially abundant between PWH-ART and HIV-1 negative controls. Remarkably, all were unknown viral species, regardless of the used pipelines. These data further highlight the need for substantial methodology improvement to better characterize the virome within the human microbiome, particularly “viral dark matter”, in order to understand its hidden role in human health conditions.^29^ At taxonomic levels above species characterized using the Phanta pipeline, one known genus, Lubbockvirus, was more abundant in PWH-ART, while Inoviridae family, Petitvirales and Tubulavirales orders, Malgrandaviricetes and Faserviricetes classes, and the Phixviricota and Hofneiviricota phyla were less abundant in PWH-ART compared to controls after adjusting for age and gender. Among these taxa, a decrease in the Inoviridae bacteriophage family has also been reported in PWH elsewhere.^16^ Inoviridae are known to infect Gram-negative bacteria without killing their host, establishing a productive chronic infection.^16^ Another study reported a higher relative abundance of Malgrandaviricetes phages in patients with chronic inflammatory bowel disease who achieved remission, linking Malgrandaviricetes to a healthier gut state.^30^ The lower abundance of these viral taxa (e.g., the Inoviridae family and Malgrandaviricetes class) may represent markers of gut dysbiosis and immunodeficiency in PWH. Possible mechanisms may include: (1) depletion of their bacterial hosts: HIV-1 infection and ART each is known to alter the gut bacteriome, when bacterial hosts decreases due to HIV-1 and ART-induced gut dysbiosis, the dependent bacteriophages (e.g., Inoviridae) may decrease; (2) gut mucosal barrier damage and enteropathy: HIV-1 targets and depletes mucosal CD4+ T cells in the gut, causing chronic inflammation and “leaky gut” syndrome (enteropathy). This disrupted and inflammatory environment may alter the physical landscape of the gut mucosa and impair the specialized microenvironments where some bacteriophages thrive; and (3) dysbiosis-driven competition: PWH have been reported to exert a marked expansion of eukaryotic viruses, e.g., Anelloviridae and Adenoviridae.^16-18^ This dramatic viral shift may create an ecological imbalance, and the expansion of eukaryotic viruses and other lytic phages (which kill their host bacteria) may consequently outcompete the chronic, slow-extruding bacteriophages such as Inoviridae. These hypotheses, however, require validation in larger and well‑matched cohorts in future studies.

Notably, our study reveals that gender influences the gut virome composition independently of HIV-1 status and age, suggesting the potential biological relevance of gender as a gut virome determinant. This finding aligns with a recent large-scale study among 4261 Chinese healthy subjects, demonstrating that the gut virome exhibits significant sex-related variations in community structure in the general population.^31^ The effect of gender on the gut virome composition is consistent with the emerging concept of the “microgenderome” – the bidirectional, sex hormone-mediated axis that shapes gut microbial ecology, immune function, and disease susceptibility.^32^ For instance, estrogens are known to bolster innate immunity and microbial diversity (e.g., enriching short-chain fatty acid-producing bacteria such as Bifidobacterium).^33^ Our finding extends the microgenderome concept to the viral component of the gut ecosystem in the context of HIV-1 infection. Moreover, the higher abundance of the Faecalibacterium viruses Toutatis and Brigit we found in males aligns with prior reports demonstrating a higher relative abundance of their likely bacterial host Faecalibacterium genus in males.^34^ ^,^ ^35^ This finding suggests that the male-predominant hormonal or physiological environment may promote the growth of Faecalibacterium bacteria, which in turn supports their specific phages (Toutatis and Brigit). Future studies investigating the interactions between the bacteriome and virome are needed to confirm this hypothesis. Moreover, the potential for hormones to directly influence bacteriophage dynamics, for example, by activating prophages integrated in the bacterial host genome via hormone-induced stress responses, remains an intriguing and unexplored research area. However, we acknowledge that in HIV cohorts, sexual behavior, particularly MSM (men who have sex with men) status, has been consistently reported to be a determinant of gut bacteriome composition.^36-38^ While direct evidence linking sexual behavior specifically to the gut virome remains limited, we cannot exclude the possibility that behavioral factors, which were unavailable in the controls in our cohort, contribute to the observed gender-associated differences in the gut virome, including the higher relative abundance of Faecalibacterium viruses Toutatis and Brigit observed in males. It is noteworthy that the effect of gender on the gut microbiome, independent of sexual behavior, is scarcely reported in the context of HIV-1 infection. For example, in studies where a *Prevotella*-dominated enterotype in MSM, as compared to a *Bacteroides*-dominated enterotype in non-MSM was consistently reported among PWH, the MSM group included only males, while non-MSM group often included both genders, and was sometimes even dominated by females.^37^ It is thus not unlikely that gender may influence both the behavioral patterns and microbial consequences in PWH. This hypothesis is supported by consistent findings that, regardless of disease state, adult males have a higher relative abundance of *Prevotella* compared to females.^34^ ^,^ ^39^ Therefore, it is possible that the *Prevotella*-dominated enterotype reported in MSM likely reflects a convergence of two effects: a baseline, hormone-driven male predisposition, which is then further amplified by the specific sexual behavior of MSM. Future studies specifically refined to account for both gender and behavioral patterns (MSM/non-MSM status for males and comparable metrics for females) are necessary to disentangle the independent contributions of biological gender *versus* sexual practices to the gut microbiome, including the virome, in PWH.

We observed correlations between certain viral taxa and clinical variables. Notably, eight vOTUs correlated inversely with both the nadir and current CD4+ T-cell counts, indicating that certain viruses – predominantly bacteriophages – may play a role in modulating host immunity, likely through intricate virome (bacteriophages)-bacteriome-host interactions. Alternatively, the alterations in host immunity due to HIV-1 infection may alter the gut bacteriome and/or virome ecology. Further studies are required to investigate the bidirectional interactions between alterations in the gut microbiome and host immunity in the context of HIV-1 infection. Intriguingly, correlations between age and viral taxa disappeared after adjusting for gender, while correlations between gender and viral taxa remained after adjusting for age, supporting that gender independently influences virome composition, and that the observed age-related correlations could be explained by gender differences. This finding highlights the importance of considering gender as a key covariate in gut virome studies. It should be noted that, due to several limitations such as the small sample size, these observed correlations warrant cautious interpretations and require validation in larger cohorts.

From a methodological perspective, our study demonstrates that virome analysis results vary considerably depending on the analytical tools and databases used. For instance, Phanta identified a substantially higher number of species-level viral taxa (4275 vOTUs) compared to MetaPhlAn4 (291 VSGs). Consequently, only six VSGs were differentially abundant between PWH-ART and controls using MetaPhlAn4, whereas a larger number of differentially abundant vOTUs (*n* = 79) were found among the viral taxa identified by Phanta. Furthermore, more significant correlations were found between clinical variables and viral taxa identified by Phanta, reflecting its greater sensitivity in capturing the full landscape of the gut virome, including potentially novel variants, in relation to clinical outcomes. These findings suggest that biologically relevant viruses and their variants may remain undetectable owing to the lack of well-curated and high-resolution viral taxonomy databases and tools. Therefore, substantial advancement in sequencing technologies, bioinformatics pipelines, and probably integrated culturomics are required to elucidate the gut virome at a more precise taxonomic level.

Furthermore, our study demonstrates that the virome diversity differed significantly at finer taxonomic levels (i.e., species and genus) between outcome groups and genders, but not at broader taxonomic levels (family, order, class, phylum). This pattern may suggest that PWH-ART‑ and gender-associated gut virome differences are manifest and detectable only at finer taxonomic resolution without altering the broader phylogenetic structure of virome communities. It should also be noted that a substantially greater number of vOTU (1993 out of 4275) were not assigned at the family level, compared to other levels (14 at genus level, 41 at order, class, and phylum level each). This aligns with the notion that over half of the gut viruses could not be assigned to known viral families via public viral database alignment,^11^ suggesting that poor viral annotations could possibly lead to underestimating the significance of the virome at a given taxonomic level. This may also explain why a few prior HIV-related virome studies, limited to family-level taxonomic resolution, reported no major change in the bacteriophage population during HIV-1 infection.

Several limitations of our study need to be addressed. First, since all PWH in this study were under ART at the time of sampling, we could not disentangle the effects of HIV-1 infection itself from potential ART-mediated influences on the observed differences in gut virome composition, the latter of which may occur indirectly via ART-induced changes in the bacterial host communities.^40^ Further studies are thus crucial to elucidate the HIV-1- and ART-mediated influences on the gut virome, and importantly the interactions between the gut virome (particular bacteriophages) and bacteriome in relation to ART and HIV-1 pathogenesis. Second, the small sample size limits statistical power, and the absence of multiple testing corrections due to the small sample size and exploratory nature of this study increases the potential risk of type I errors; thus, our findings are hypothesis-generating and require validation in larger cohorts. Third, owing to the lack of sexual behavior information in the controls and only one MSM in the PWH-ART group, we were unable to distinguish whether the observed gender effect on the gut virome was driven by biological gender or by behavioral patterns that correlate with it, or by both. Future studies should incorporate both gender and detailed behavioral phenotyping (e.g., MSM/non-MSM status) to disentangle the independent contributions of biological sex versus sexual behavior to the gut virome in PWH. Fourth, a trade-off between the sensitivity and specificity of virus detection was necessary. Consequently, viruses present at low concentrations, such as eukaryotic viruses, may have been omitted from our analysis. However, given that a consistent pattern of eukaryotic virome alterations has already been established in HIV-1 infection,^16-18^ herein, we focused on the prokaryotic DNA virome – the dominant yet poorly characterized viral component – where prior family-level analyses have reported no major changes. Indeed, the absence of significant changes in bacteriophage families in our study aligns with these earlier reports. This finding underscores that high-resolution virome profiling is crucial to uncover finer gut virome signatures in HIV-1 infection – signatures that remain obscured when taxonomic resolution is limited to the family level or higher. Moreover, virome characterization in general is hampered by challenges such as the lack of standardized high-resolution taxonomic databases and standardized bioinformatics pipelines.^4^ Consequently, study results can vary dramatically using different databases and tools; thus, comparisons across studies and data interpretations must be made cautiously. Additionally, most viruses – including those differentially abundant in PWH‑ART – remain unclassified at the species and genus levels, posing a fundamental challenge to the clinical translation of research findings. Nevertheless, from the outset, our primary goal was to perform an exploratory study and evaluate the existing virome pipelines to establish whether a large-scale and comprehensive study is justified. Our exploratory data strongly indicate that such a comprehensive investigation is indeed warranted to understand the correlations between the gut virome and HIV-1 infection with and without ART, and to further explore the mechanisms and potential role of the gut virome in HIV-1 pathogenesis.

In conclusion, our study unravels significant differences in gut prokaryotic DNA virome composition, characterized by different beta diversity and differential abundance of individual viral taxa, between PWH-ART and HIV-1 negative individuals. Remarkably, all the differentially abundant viral taxa were undescribed viral species. Correlations were found between clinical variables and certain viral taxa of undescribed species. Furthermore, gender appears to be an independent factor influencing the gut DNA virome composition. From the perspective of methodological assessment, our study underscores the importance of further development of sequencing technologies, reference databases, and bioinformatics tools to precisely characterize the gut virome and to elucidate the “viral dark matter” in the context of human health and disease.

## Supplementary Material

Supplementary Material Supplementary_Materials_proofed_15_Jun_2026_15_12_AU.docx

## Data Availability

The raw shotgun metagenome sequencing data have been submitted to GenBank Sequence Read Archive under the BioProject ID PRJNA692830.
