# Supplementary material for: Species-level virome profiling reveals compositional differences in the gut prokaryotic DNA virome of people with HIV-1 on antiretroviral therapy
Source: Gut Microbes Rep. 2026 Jun 18;3(1):2688064. doi: 10.1080/29933935.2026.2688064 (PMC13285549; doi:10.1080/29933935.2026.2688064)
Supplement: Supplementary Material — Supplementary_Materials_proofed_15_Jun_2026_15_12_AU.docx [file KGMR_A_2688064_SM3040.docx]

**Supplementary Materials**

**
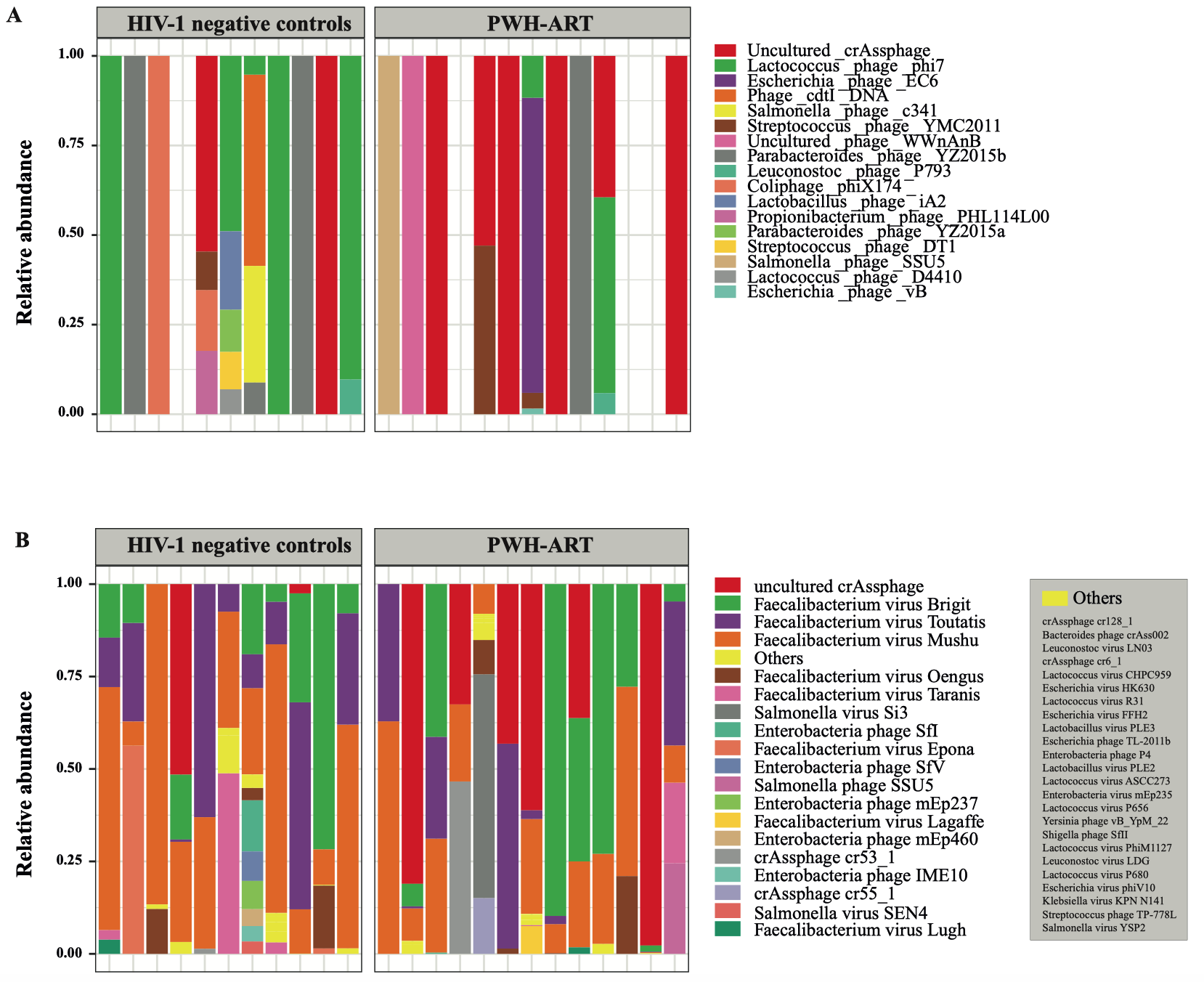
**

**Figure S1.** Composition and relative abundance of known viral species identified using MetaPhlAn4 pipeline (**A**) and Phanta pipeline (**B**). MetaPhlAn4 identified 17 viral sequence groups (VSGs) of known species (A). Phanta identified 43 species‑level viral Operational Taxonomic Units (vOTUs) of known species, the top 19 are shown individually, and the remaining 24 are grouped as "Others" with individual names being indicated in the legend (B).

**
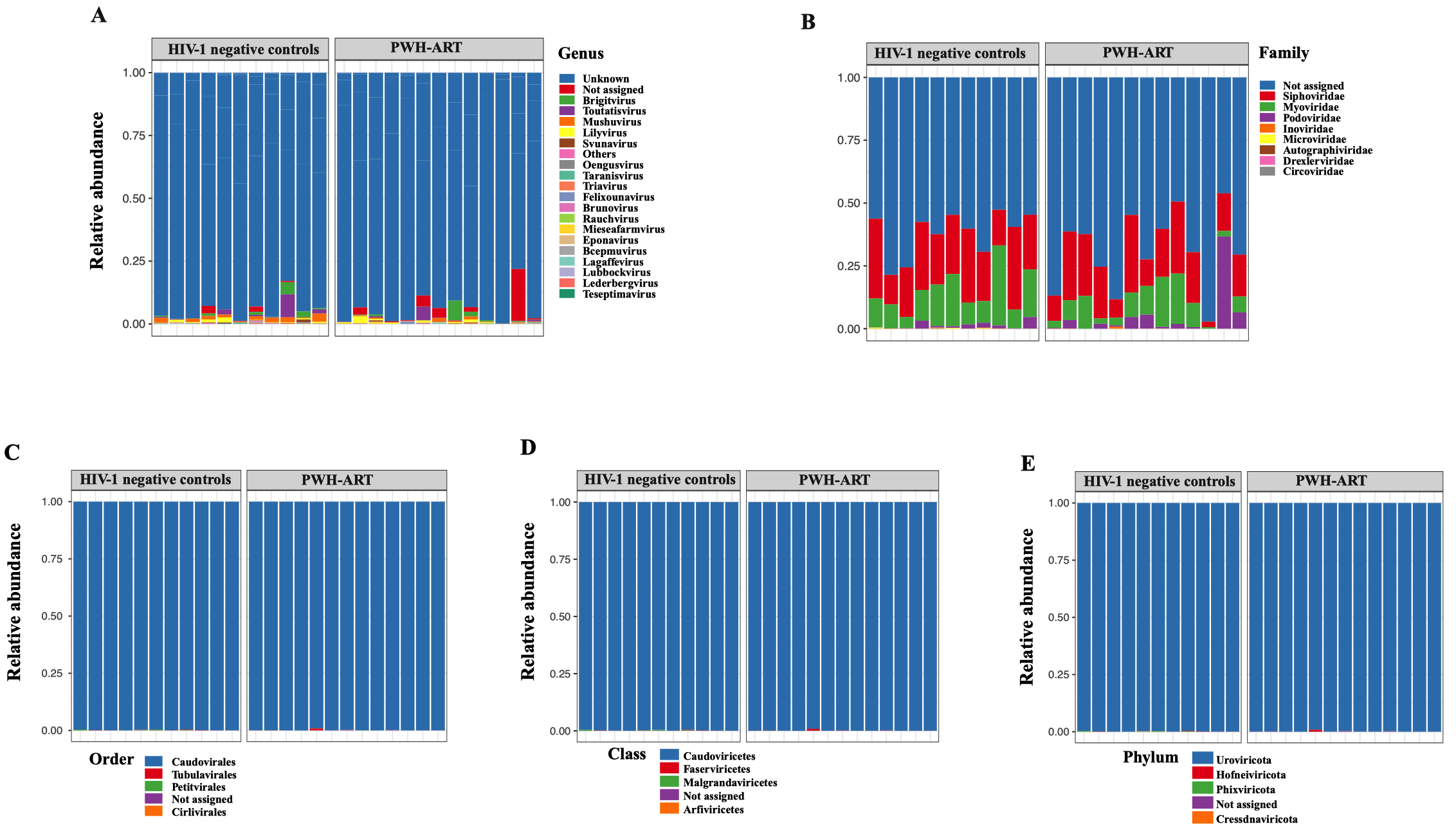
**

**Figure S2.** Composition and relative abundance of viral taxa identified using Phanta pipeline with its default database, at genus (**A**), family (**B**), order (**C**), class (**D**), and phylum (**E**) level. Taxa are ordered by their decreasing total relative abundance across samples. For genus level (A), top 18 of 34 known viral genera are indicated. ´Others´ corresponds to remaining 16 known viral genera. ´Unknown´ indicates sequences that can be assigned to unknown taxa at genus level, while ´Not assigned´ indicates sequences that cannot be assigned to genus level. For other taxonomic levels (B-E), all identified viral taxa at a given taxonomic level are shown, ´Not assigned´ indicates sequences that cannot be assigned to a given taxonomic level.

**Table S1.** Differentially abundant viruses at species level between outcome groups (PWH-ART and controls) assigned by MetaPhlAn4 (**A**) and Phanta (**B**), and between genders (males and females) assigned by MetaPhlAn4 (**C**) and Phanta (**D**), and their taxonomy details. Differential abundance analysis was performed using ANCOM-BC, adjusted for covariates (outcome group adjusted for age and gender; gender adjusted for outcome group and age). Taxa with Log2 fold change>0 are more abundant in PWH-ART and males, as compared to controls and females, respectively. Taxa with Log2 fold change<0 are less abundant in PWH-ART and males, as compared to controls and females, respectively. Taxa with p<0.1 are presented in the Table. Statistically significantly differential abundant taxa with p<0.05 are highlighted in green.

**A**

| **Taxon** | **Log2 Fold Change** | **p value** | **Taxonomy detail** |
| --- | --- | --- | --- |
| M914 | -0.01710 | 0.00182 | unknown |
| M697 | -0.13464 | 0.01007 | unknown |
| M892 | -0.05366 | 0.01058 | unknown |
| M460 | -0.03081 | 0.01396 | unknown |
| M459 | -0.01383 | 0.02697 | unknown |
| M756 | -0.02470 | 0.03272 | unknown |
| M911 | 0.06198 | 0.06043 | unknown |
| M1106 | 0.00517 | 0.06228 | unknown |
| M936 | -0.00313 | 0.07173 | unknown |
| M1147 | -0.00314 | 0.07348 | unknown |

**B**

| **Taxon** | **Log2 Fold Change** | **p value** | **Taxonomy details** |
| --- | --- | --- | --- |
| OTU-02174 | 7.75379 | 1.98E-14 | phylum_Uroviricota\|class_Caudoviricetes\|order_Caudovirales\|family_Myoviridae\|genus_mgv_g_4000073\|species_OTU-02174 |
| OTU-52713 | -5.94379 | 1.63E-09 | phylum_Uroviricota\|class_Caudoviricetes\|order_Caudovirales\|genus_mgv_g_4000108\|species_OTU-52713 |
| OTU-61473 | 9.78384 | 1.87E-08 | phylum_Uroviricota\|class_Caudoviricetes\|order_Caudovirales\|genus_mgv_g_4000005\|species_OTU-61473 |
| OTU-29582 | -5.46031 | 1.90E-06 | phylum_Uroviricota\|class_Caudoviricetes\|order_Caudovirales\|genus_mgv_g_4000399\|species_OTU-29582 |
| OTU-71865 | -5.31395 | 1.20E-05 | phylum_Uroviricota\|class_Caudoviricetes\|order_Caudovirales\|genus_mgv_g_4000005\|species_OTU-71865 |
| OTU-65781 | -3.89992 | 1.28E-04 | phylum_Uroviricota\|class_Caudoviricetes\|order_Caudovirales\|family_Siphoviridae\|genus_mgv_g_4000371\|species_OTU-65781 |
| OTU-20465 | -4.94723 | 1.45E-04 | phylum_Uroviricota\|class_Caudoviricetes\|order_Caudovirales\|genus_mgv_g_4000503\|species_OTU-20465 |
| OTU-13587 | -4.28964 | 7.59E-04 | phylum_Uroviricota\|class_Caudoviricetes\|order_Caudovirales\|family_Siphoviridae\|genus_mgv_g_4000155\|species_OTU-13587 |
| OTU-66940 | -4.23674 | 1.22E-03 | phylum_Uroviricota\|class_Caudoviricetes\|order_Caudovirales\|genus_mgv_g_4000016\|species_OTU-66940 |
| OTU-50196 | -4.51221 | 2.25E-03 | phylum_Uroviricota\|class_Caudoviricetes\|order_Caudovirales\|genus_mgv_g_4000108\|species_OTU-50196 |
| OTU-68119 | -5.43574 | 2.30E-03 | phylum_Uroviricota\|class_Caudoviricetes\|order_Caudovirales\|family_Siphoviridae\|genus_mgv_g_4000673\|species_OTU-68119 |
| OTU-49760 | -5.28404 | 2.83E-03 | phylum_Uroviricota\|class_Caudoviricetes\|order_Caudovirales\|genus_mgv_g_4000224\|species_OTU-49760 |
| OTU-32458 | -5.09367 | 2.99E-03 | phylum_Uroviricota\|class_Caudoviricetes\|order_Caudovirales\|family_Siphoviridae\|genus_mgv_g_4000162\|species_OTU-32458 |
| OTU-67435 | 5.62264 | 3.05E-03 | phylum_Uroviricota\|class_Caudoviricetes\|order_Caudovirales\|genus_mgv_g_4001074\|species_OTU-67435 |
| OTU-02076 | -3.45915 | 3.47E-03 | phylum_Uroviricota\|class_Caudoviricetes\|order_Caudovirales\|genus_mgv_g_4000373\|species_OTU-02076 |
| OTU-44956 | -3.33030 | 3.62E-03 | phylum_Uroviricota\|class_Caudoviricetes\|order_Caudovirales\|genus_mgv_g_4000069\|species_OTU-44956 |
| OTU-35846 | -4.81574 | 4.11E-03 | phylum_Uroviricota\|class_Caudoviricetes\|order_Caudovirales\|genus_mgv_g_4000221\|species_OTU-35846 |
| OTU-18637 | -3.23437 | 4.97E-03 | phylum_Uroviricota\|class_Caudoviricetes\|order_Caudovirales\|genus_Lilyvirus\|species_OTU-18637 |
| OTU-73639 | 4.36688 | 5.08E-03 | phylum_Uroviricota\|class_Caudoviricetes\|order_Caudovirales\|genus_mgv_g_4000213\|species_OTU-73639 |
| OTU-08071 | -4.15620 | 5.55E-03 | phylum_Uroviricota\|class_Caudoviricetes\|order_Caudovirales\|genus_mgv_g_4000016\|species_OTU-08071 |
| OTU-05987 | -3.04315 | 5.72E-03 | phylum_Uroviricota\|class_Caudoviricetes\|order_Caudovirales\|genus_mgv_g_4000373\|species_OTU-05987 |
| OTU-72982 | -4.06671 | 7.02E-03 | phylum_Uroviricota\|class_Caudoviricetes\|order_Caudovirales\|genus_mgv_g_4000095\|species_OTU-72982 |
| OTU-32886 | -5.97213 | 7.08E-03 | phylum_Uroviricota\|class_Caudoviricetes\|order_Caudovirales\|genus_mgv_g_4000016\|species_OTU-32886 |
| OTU-31534 | -3.37804 | 7.87E-03 | phylum_Uroviricota\|class_Caudoviricetes\|order_Caudovirales\|family_Myoviridae\|genus_mgv_g_4000148\|species_OTU-31534 |
| OTU-47793 | -5.93768 | 8.11E-03 | phylum_Uroviricota\|class_Caudoviricetes\|order_Caudovirales\|genus_mgv_g_4000016\|species_OTU-47793 |
| OTU-70236 | -4.12824 | 9.13E-03 | phylum_Uroviricota\|class_Caudoviricetes\|order_Caudovirales\|genus_mgv_g_4000005\|species_OTU-70236 |
| OTU-09469 | 3.82274 | 9.16E-03 | phylum_Uroviricota\|class_Caudoviricetes\|order_Caudovirales\|genus_mgv_g_4001693\|species_OTU-09469 |
| OTU-56912 | 4.52574 | 9.92E-03 | phylum_Uroviricota\|class_Caudoviricetes\|order_Caudovirales\|family_Siphoviridae\|genus_mgv_g_4000018\|species_OTU-56912 |
| OTU-74571 | 4.54113 | 9.97E-03 | phylum_Uroviricota\|class_Caudoviricetes\|order_Caudovirales\|family_Siphoviridae\|genus_mgv_g_4000018\|species_OTU-74571 |
| OTU-51113 | -3.21544 | 1.02E-02 | phylum_Uroviricota\|class_Caudoviricetes\|order_Caudovirales\|family_Siphoviridae\|genus_mgv_g_4000018\|species_OTU-51113 |
| OTU-17531 | 4.65062 | 1.09E-02 | phylum_Uroviricota\|class_Caudoviricetes\|order_Caudovirales\|genus_mgv_g_4000324\|species_OTU-17531 |
| OTU-25163 | 4.86777 | 1.20E-02 | phylum_Uroviricota\|class_Caudoviricetes\|order_Caudovirales\|family_Siphoviridae\|genus_mgv_g_4000173\|species_OTU-25163 |
| OTU-17994 | 1.77359 | 1.22E-02 | phylum_Uroviricota\|class_Caudoviricetes\|order_Caudovirales\|genus_mgv_g_4001256\|species_OTU-17994 |
| OTU-05722 | -3.75888 | 1.32E-02 | phylum_Uroviricota\|class_Caudoviricetes\|order_Caudovirales\|family_Myoviridae\|genus_mgv_g_4002261\|species_OTU-05722 |
| OTU-06403 | 4.56576 | 1.36E-02 | phylum_Uroviricota\|class_Caudoviricetes\|order_Caudovirales\|family_Siphoviridae\|genus_mgv_g_4000419\|species_OTU-06403 |
| OTU-45424 | 2.92146 | 1.40E-02 | phylum_Uroviricota\|class_Caudoviricetes\|order_Caudovirales\|family_Myoviridae\|genus_mgv_g_4000251\|species_OTU-45424 |
| OTU-23520 | -3.07272 | 1.41E-02 | phylum_Uroviricota\|class_Caudoviricetes\|order_Caudovirales\|genus_mgv_g_4000092\|species_OTU-23520 |
| OTU-30083 | 2.32628 | 1.46E-02 | phylum_Uroviricota\|class_Caudoviricetes\|order_Caudovirales\|family_Siphoviridae\|genus_mgv_g_4000001\|species_OTU-30083 |
| OTU-19699 | -3.80072 | 1.47E-02 | phylum_Uroviricota\|class_Caudoviricetes\|order_Caudovirales\|genus_mgv_g_4000016\|species_OTU-19699 |
| OTU-57052 | -3.22065 | 1.48E-02 | phylum_Uroviricota\|class_Caudoviricetes\|order_Caudovirales\|genus_mgv_g_4000085\|species_OTU-57052 |
| OTU-62859 | 6.61727 | 1.49E-02 | phylum_Uroviricota\|class_Caudoviricetes\|order_Caudovirales\|genus_mgv_g_4000040\|species_OTU-62859 |
| OTU-31483 | 3.93767 | 1.61E-02 | phylum_Uroviricota\|class_Caudoviricetes\|order_Caudovirales\|genus_mgv_g_4000812\|species_OTU-31483 |
| OTU-46395 | 4.82533 | 1.67E-02 | phylum_Uroviricota\|class_Caudoviricetes\|order_Caudovirales\|family_Siphoviridae\|genus_mgv_g_4000096\|species_OTU-46395 |
| OTU-17762 | -4.28388 | 1.72E-02 | phylum_Uroviricota\|class_Caudoviricetes\|order_Caudovirales\|genus_mgv_g_4000248\|species_OTU-17762 |
| OTU-45276 | 3.43449 | 1.85E-02 | phylum_Uroviricota\|class_Caudoviricetes\|order_Caudovirales\|family_Siphoviridae\|genus_mgv_g_4000673\|species_OTU-45276 |
| OTU-17810 | -3.89577 | 2.01E-02 | phylum_Uroviricota\|class_Caudoviricetes\|order_Caudovirales\|family_Myoviridae\|genus_mgv_g_4000073\|species_OTU-17810 |
| OTU-45085 | 3.73953 | 2.16E-02 | phylum_Uroviricota\|class_Caudoviricetes\|order_Caudovirales\|family_Siphoviridae\|genus_mgv_g_4000018\|species_OTU-45085 |
| OTU-69103 | 3.68318 | 2.17E-02 | phylum_Uroviricota\|class_Caudoviricetes\|order_Caudovirales\|family_Siphoviridae\|genus_mgv_g_4000018\|species_OTU-69103 |
| OTU-25852 | 4.03170 | 2.29E-02 | phylum_Uroviricota\|class_Caudoviricetes\|order_Caudovirales\|family_Siphoviridae\|genus_mgv_g_4000483\|species_OTU-25852 |
| OTU-48794 | 3.72347 | 2.32E-02 | phylum_Uroviricota\|class_Caudoviricetes\|order_Caudovirales\|genus_mgv_g_4000899\|species_OTU-48794 |
| OTU-25897 | -3.75767 | 2.35E-02 | phylum_Uroviricota\|class_Caudoviricetes\|order_Caudovirales\|genus_mgv_g_4000291\|species_OTU-25897 |
| OTU-41754 | -3.35311 | 2.47E-02 | phylum_Uroviricota\|class_Caudoviricetes\|order_Caudovirales\|family_Siphoviridae\|genus_mgv_g_4000371\|species_OTU-41754 |
| OTU-36944 | 3.00318 | 2.50E-02 | phylum_Uroviricota\|class_Caudoviricetes\|order_Caudovirales\|genus_mgv_g_4000865\|species_OTU-36944 |
| OTU-67046 | 5.80079 | 2.62E-02 | phylum_Uroviricota\|class_Caudoviricetes\|order_Caudovirales\|genus_mgv_g_4000092\|species_OTU-67046 |
| OTU-43874 | -3.79108 | 2.75E-02 | phylum_Uroviricota\|class_Caudoviricetes\|order_Caudovirales\|genus_mgv_g_4000032\|species_OTU-43874 |
| OTU-65474 | -3.32721 | 2.97E-02 | phylum_Uroviricota\|class_Caudoviricetes\|order_Caudovirales\|genus_mgv_g_4000301\|species_OTU-65474 |
| OTU-52870 | -3.21252 | 3.01E-02 | phylum_Uroviricota\|class_Caudoviricetes\|order_Caudovirales\|family_Siphoviridae\|genus_mgv_g_4000004\|species_OTU-52870 |
| OTU-57488 | 3.68437 | 3.02E-02 | phylum_Uroviricota\|class_Caudoviricetes\|order_Caudovirales\|family_Siphoviridae\|genus_mgv_g_4000146\|species_OTU-57488 |
| OTU-25613 | -3.48863 | 3.11E-02 | phylum_Uroviricota\|class_Caudoviricetes\|order_Caudovirales\|genus_mgv_g_4001919\|species_OTU-25613 |
| OTU-29182 | -2.71595 | 3.21E-02 | phylum_Uroviricota\|class_Caudoviricetes\|order_Caudovirales\|genus_mgv_g_4000274\|species_OTU-29182 |
| OTU-54271 | 5.33130 | 3.22E-02 | phylum_Uroviricota\|class_Caudoviricetes\|order_Caudovirales\|genus_mgv_g_4000504\|species_OTU-54271 |
| OTU-12199 | -3.08115 | 3.22E-02 | phylum_Uroviricota\|class_Caudoviricetes\|order_Caudovirales\|family_Siphoviridae\|genus_mgv_g_4000001\|species_OTU-12199 |
| OTU-24221 | -4.41091 | 3.27E-02 | phylum_Uroviricota\|class_Caudoviricetes\|order_Caudovirales\|family_Siphoviridae\|genus_mgv_g_4000001\|species_OTU-24221 |
| OTU-53049 | -3.27066 | 3.41E-02 | phylum_Uroviricota\|class_Caudoviricetes\|order_Caudovirales\|family_Siphoviridae\|genus_mgv_g_4000147\|species_OTU-53049 |
| OTU-54621 | 2.24278 | 3.51E-02 | genus_mgv_g_4000215\|species_OTU-54621 |
| OTU-56282 | -4.08762 | 3.57E-02 | phylum_Uroviricota\|class_Caudoviricetes\|order_Caudovirales\|genus_mgv_g_4000026\|species_OTU-56282 |
| OTU-68468 | -4.01775 | 3.59E-02 | phylum_Uroviricota\|class_Caudoviricetes\|order_Caudovirales\|genus_mgv_g_4000005\|species_OTU-68468 |
| OTU-02762 | 4.67340 | 3.61E-02 | phylum_Uroviricota\|class_Caudoviricetes\|order_Caudovirales\|genus_mgv_g_4000545\|species_OTU-02762 |
| OTU-41039 | 2.90461 | 3.62E-02 | phylum_Uroviricota\|class_Caudoviricetes\|order_Caudovirales\|genus_mgv_g_4000722\|species_OTU-41039 |
| OTU-56689 | -2.56426 | 3.72E-02 | phylum_Uroviricota\|class_Caudoviricetes\|order_Caudovirales\|genus_mgv_g_4002814\|species_OTU-56689 |
| OTU-17654 | 6.03877 | 3.75E-02 | phylum_Uroviricota\|class_Caudoviricetes\|order_Caudovirales\|genus_mgv_g_4000005\|species_OTU-17654 |
| OTU-38768 | 3.24475 | 3.89E-02 | phylum_Uroviricota\|class_Caudoviricetes\|order_Caudovirales\|family_Siphoviridae\|genus_mgv_g_4000338\|species_OTU-38768 |
| OTU-66065 | -2.56691 | 4.14E-02 | phylum_Uroviricota\|class_Caudoviricetes\|order_Caudovirales\|genus_mgv_g_4000032\|species_OTU-66065 |
| OTU-34487 | -3.92734 | 4.18E-02 | phylum_Uroviricota\|class_Caudoviricetes\|order_Caudovirales\|family_Siphoviridae\|genus_mgv_g_4000007\|species_OTU-34487 |
| OTU-15362 | -3.43925 | 4.20E-02 | phylum_Uroviricota\|class_Caudoviricetes\|order_Caudovirales\|family_Myoviridae\|genus_mgv_g_4000780\|species_OTU-15362 |
| OTU-60056 | 4.36248 | 4.40E-02 | phylum_Uroviricota\|class_Caudoviricetes\|order_Caudovirales\|genus_mgv_g_4000223\|species_OTU-60056 |
| OTU-72669 | 3.94818 | 4.57E-02 | phylum_Uroviricota\|class_Caudoviricetes\|order_Caudovirales\|genus_mgv_g_4000022\|species_OTU-72669 |
| OTU-46073 | 2.51648 | 4.90E-02 | phylum_Uroviricota\|class_Caudoviricetes\|order_Caudovirales\|genus_mgv_g_4000016\|species_OTU-46073 |
| OTU-53052 | 2.98180 | 4.92E-02 | phylum_Uroviricota\|class_Caudoviricetes\|order_Caudovirales\|genus_mgv_g_4001406\|species_OTU-53052 |
| OTU-16387 | -2.95010 | 5.17E-02 | phylum_Uroviricota\|class_Caudoviricetes\|order_Caudovirales\|genus_mgv_g_4000005\|species_OTU-16387 |
| OTU-40484 | 3.28197 | 5.31E-02 | phylum_Uroviricota\|class_Caudoviricetes\|order_Caudovirales\|family_Siphoviridae\|genus_mgv_g_4000018\|species_OTU-40484 |
| OTU-58724 | 3.82299 | 5.43E-02 | phylum_Uroviricota\|class_Caudoviricetes\|order_Caudovirales\|genus_mgv_g_4000020\|species_OTU-58724 |
| OTU-16922 | 1.56694 | 5.44E-02 | phylum_Uroviricota\|class_Caudoviricetes\|order_Caudovirales\|genus_mgv_g_4000896\|species_OTU-16922 |
| OTU-18399 | -2.64802 | 5.58E-02 | phylum_Uroviricota\|class_Caudoviricetes\|order_Caudovirales\|family_Siphoviridae\|genus_mgv_g_4000286\|species_OTU-18399 |
| OTU-21954 | -2.15704 | 5.78E-02 | phylum_Uroviricota\|class_Caudoviricetes\|order_Caudovirales\|genus_mgv_g_4000075\|species_OTU-21954 |
| OTU-44307 | -3.51178 | 6.24E-02 | phylum_Uroviricota\|class_Caudoviricetes\|order_Caudovirales\|genus_mgv_g_4000118\|species_OTU-44307 |
| OTU-33561 | -2.39393 | 6.33E-02 | phylum_Uroviricota\|class_Caudoviricetes\|order_Caudovirales\|genus_mgv_g_4000189\|species_OTU-33561 |
| OTU-07521 | -1.93557 | 6.40E-02 | phylum_Hofneiviricota\|class_Faserviricetes\|order_Tubulavirales\|family_Inoviridae\|genus_mgv_g_4000202\|species_OTU-07521 |
| OTU-16669 | 1.34306 | 6.93E-02 | phylum_Uroviricota\|class_Caudoviricetes\|order_Caudovirales\|family_Siphoviridae\|genus_mgv_g_4000196\|species_OTU-16669 |
| OTU-27128 | -3.53540 | 7.01E-02 | phylum_Uroviricota\|class_Caudoviricetes\|order_Caudovirales\|genus_mgv_g_4000016\|species_OTU-27128 |
| OTU-50046 | -2.69144 | 7.15E-02 | phylum_Uroviricota\|class_Caudoviricetes\|order_Caudovirales\|family_Siphoviridae\|genus_mgv_g_4000001\|species_OTU-50046 |
| OTU-72263 | 3.15924 | 7.15E-02 | phylum_Uroviricota\|class_Caudoviricetes\|order_Caudovirales\|family_Siphoviridae\|genus_mgv_g_4000018\|species_OTU-72263 |
| OTU-68238 | 1.92353 | 7.17E-02 | phylum_Uroviricota\|class_Caudoviricetes\|order_Caudovirales\|family_Myoviridae\|genus_mgv_g_4000273\|species_OTU-68238 |
| OTU-46698 | 1.32427 | 7.18E-02 | phylum_Uroviricota\|class_Caudoviricetes\|order_Caudovirales\|family_Siphoviridae\|genus_mgv_g_4000001\|species_OTU-46698 |
| OTU-22248 | -1.96350 | 7.24E-02 | phylum_Uroviricota\|class_Caudoviricetes\|order_Caudovirales\|family_Myoviridae\|genus_mgv_g_4000148\|species_OTU-22248 |
| OTU-40825 | -2.13619 | 7.30E-02 | phylum_Uroviricota\|class_Caudoviricetes\|order_Caudovirales\|family_Myoviridae\|genus_mgv_g_4000073\|species_OTU-40825 |
| OTU-56362 | -2.17033 | 7.47E-02 | phylum_Uroviricota\|class_Caudoviricetes\|order_Caudovirales\|genus_mgv_g_4000016\|species_OTU-56362 |
| OTU-76700 | 2.51811 | 7.47E-02 | phylum_Uroviricota\|class_Caudoviricetes\|order_Caudovirales\|family_Siphoviridae\|genus_mgv_g_4000181\|species_OTU-76700 |
| OTU-30370 | 3.38922 | 7.62E-02 | phylum_Uroviricota\|class_Caudoviricetes\|order_Caudovirales\|family_Siphoviridae\|genus_mgv_g_4000419\|species_OTU-30370 |
| OTU-35079 | 3.54056 | 7.78E-02 | phylum_Uroviricota\|class_Caudoviricetes\|order_Caudovirales\|genus_mgv_g_4000095\|species_OTU-35079 |
| OTU-31703 | 0.98682 | 7.93E-02 | phylum_Uroviricota\|class_Caudoviricetes\|order_Caudovirales\|genus_mgv_g_4000005\|species_OTU-31703 |
| OTU-35365 | 1.94696 | 7.97E-02 | phylum_Uroviricota\|class_Caudoviricetes\|order_Caudovirales\|genus_mgv_g_4000228\|species_OTU-35365 |
| OTU-14225 | 2.84180 | 7.98E-02 | phylum_Uroviricota\|class_Caudoviricetes\|order_Caudovirales\|family_Siphoviridae\|genus_mgv_g_4000320\|species_OTU-14225 |
| OTU-05170 | 4.09823 | 8.14E-02 | phylum_Uroviricota\|class_Caudoviricetes\|order_Caudovirales\|genus_mgv_g_4000022\|species_OTU-05170 |
| OTU-71288 | -2.04181 | 8.20E-02 | phylum_Uroviricota\|class_Caudoviricetes\|order_Caudovirales\|family_Siphoviridae\|genus_mgv_g_4000001\|species_OTU-71288 |
| OTU-00428 | 1.79878 | 8.20E-02 | phylum_Uroviricota\|class_Caudoviricetes\|order_Caudovirales\|genus_mgv_g_4002440\|species_OTU-00428 |
| OTU-02838 | -1.96702 | 8.56E-02 | phylum_Uroviricota\|class_Caudoviricetes\|order_Caudovirales\|genus_mgv_g_4000213\|species_OTU-02838 |
| OTU-32387 | 2.47107 | 8.61E-02 | phylum_Uroviricota\|class_Caudoviricetes\|order_Caudovirales\|family_Siphoviridae\|genus_mgv_g_4000885\|species_OTU-32387 |
| OTU-37393 | 2.81899 | 8.63E-02 | phylum_Uroviricota\|class_Caudoviricetes\|order_Caudovirales\|genus_mgv_g_4000068\|species_OTU-37393 |
| OTU-38558 | -1.50832 | 8.63E-02 | phylum_Uroviricota\|class_Caudoviricetes\|order_Caudovirales\|family_Myoviridae\|genus_mgv_g_4001069\|species_OTU-38558 |
| OTU-38589 | 2.68019 | 8.86E-02 | phylum_Uroviricota\|class_Caudoviricetes\|order_Caudovirales\|family_Siphoviridae\|genus_mgv_g_4000093\|species_OTU-38589 |
| OTU-62258 | 2.49589 | 8.88E-02 | phylum_Uroviricota\|class_Caudoviricetes\|order_Caudovirales\|family_Siphoviridae\|genus_mgv_g_4000083\|species_OTU-62258 |
| OTU-03817 | 3.22897 | 8.90E-02 | phylum_Uroviricota\|class_Caudoviricetes\|order_Caudovirales\|family_Myoviridae\|genus_mgv_g_4000062\|species_OTU-03817 |
| OTU-55784 | -3.48942 | 9.00E-02 | phylum_Uroviricota\|class_Caudoviricetes\|order_Caudovirales\|family_Siphoviridae\|genus_mgv_g_4000001\|species_OTU-55784 |
| OTU-63879 | -2.12665 | 9.41E-02 | phylum_Uroviricota\|class_Caudoviricetes\|order_Caudovirales\|genus_mgv_g_4001066\|species_OTU-63879 |
| OTU-14917 | -1.89369 | 9.64E-02 | phylum_Uroviricota\|class_Caudoviricetes\|order_Caudovirales\|genus_mgv_g_4000016\|species_OTU-14917 |
| OTU-30855 | 2.26741 | 9.78E-02 | phylum_Uroviricota\|class_Caudoviricetes\|order_Caudovirales\|family_Myoviridae\|genus_Brunovirus\|species_OTU-30855 |
| OTU-01640 | 2.27340 | 9.95E-02 | phylum_Uroviricota\|class_Caudoviricetes\|order_Caudovirales\|family_Siphoviridae\|genus_mgv_g_4000021\|species_OTU-01640 |
| OTU-14110 | 3.43607 | 9.97E-02 | phylum_Uroviricota\|class_Caudoviricetes\|order_Caudovirales\|family_Siphoviridae\|genus_mgv_g_4000927\|species_OTU-14110 |

**C**

| **Taxon** | **Log2 Fold Change** | **p value** | **Taxonomy detail** |
| --- | --- | --- | --- |
| M506 | -0.06388 | 0.02750 | unknown |
| M282 | 0.09410 | 0.03265 | unknown |
| M488 | -0.04400 | 0.04618 | unknown |
| M697 | -0.13018 | 0.04944 | unknown |
| M801 | -0.04022 | 0.05281 | unknown |
| M911 | 0.05540 | 0.05779 | unknown |
| M229 | 0.04067 | 0.05922 | unknown |
| M1140 | -0.05086 | 0.06912 | unknown |

**D**

| **Taxon** | **Log2 Fold Change** | **p value** | **Taxonomy detail** |
| --- | --- | --- | --- |
| OTU-02174 | 7.5041 | 8.47E-11 | phylum_Uroviricota\|class_Caudoviricetes\|order_Caudovirales\|family_Myoviridae\|genus_mgv_g_4000073\|species_OTU-02174 |
| OTU-75628 | 5.8764 | 5.14E-04 | phylum_Uroviricota\|class_Caudoviricetes\|order_Caudovirales\|family_Siphoviridae\|genus_mgv_g_4000001\|species_OTU-75628 |
| Faecalibacterium virus Toutatis | 4.1920 | 6.81E-04 | phylum_Uroviricota\|class_Caudoviricetes\|order_Caudovirales\|family_Myoviridae\|genus_Toutatisvirus\|species_Faecalibacterium virus Toutatis |
| OTU-01501 | -4.5600 | 8.50E-04 | phylum_Uroviricota\|class_Caudoviricetes\|order_Caudovirales\|genus_mgv_g_4000016\|species_OTU-01501 |
| OTU-21850 | -3.5065 | 9.45E-04 | phylum_Uroviricota\|class_Caudoviricetes\|order_Caudovirales\|genus_mgv_g_4000289\|species_OTU-21850 |
| OTU-74628 | -5.4277 | 1.53E-03 | phylum_Uroviricota\|class_Caudoviricetes\|order_Caudovirales\|genus_mgv_g_4000016\|species_OTU-74628 |
| OTU-73639 | 4.3497 | 3.18E-03 | phylum_Uroviricota\|class_Caudoviricetes\|order_Caudovirales\|genus_mgv_g_4000213\|species_OTU-73639 |
| Faecalibacterium virus Brigit | 5.1994 | 3.85E-03 | phylum_Uroviricota\|class_Caudoviricetes\|order_Caudovirales\|family_Myoviridae\|genus_Brigitvirus\|species_Faecalibacterium virus Brigit |
| OTU-61473 | 6.7523 | 4.06E-03 | phylum_Uroviricota\|class_Caudoviricetes\|order_Caudovirales\|genus_mgv_g_4000005\|species_OTU-61473 |
| OTU-56912 | 4.7382 | 4.32E-03 | phylum_Uroviricota\|class_Caudoviricetes\|order_Caudovirales\|family_Siphoviridae\|genus_mgv_g_4000018\|species_OTU-56912 |
| OTU-72263 | 5.2505 | 4.59E-03 | phylum_Uroviricota\|class_Caudoviricetes\|order_Caudovirales\|family_Siphoviridae\|genus_mgv_g_4000018\|species_OTU-72263 |
| OTU-20465 | -3.7601 | 4.63E-03 | phylum_Uroviricota\|class_Caudoviricetes\|order_Caudovirales\|genus_mgv_g_4000503\|species_OTU-20465 |
| OTU-71865 | -3.3600 | 5.99E-03 | phylum_Uroviricota\|class_Caudoviricetes\|order_Caudovirales\|genus_mgv_g_4000005\|species_OTU-71865 |
| OTU-37502 | 3.4022 | 6.37E-03 | phylum_Uroviricota\|class_Caudoviricetes\|order_Caudovirales\|genus_mgv_g_4000213\|species_OTU-37502 |
| OTU-58532 | 2.4268 | 7.37E-03 | phylum_Uroviricota\|class_Caudoviricetes\|order_Caudovirales\|family_Siphoviridae\|genus_mgv_g_4000231\|species_OTU-58532 |
| OTU-40898 | 4.7982 | 7.44E-03 | phylum_Uroviricota\|class_Caudoviricetes\|order_Caudovirales\|genus_mgv_g_4000022\|species_OTU-40898 |
| OTU-34604 | -2.8798 | 7.46E-03 | phylum_Uroviricota\|class_Caudoviricetes\|order_Caudovirales\|family_Siphoviridae\|genus_mgv_g_4000196\|species_OTU-34604 |
| OTU-39788 | -3.6708 | 7.74E-03 | phylum_Uroviricota\|class_Caudoviricetes\|order_Caudovirales\|genus_mgv_g_4000016\|species_OTU-39788 |
| OTU-65104 | -4.0771 | 9.15E-03 | phylum_Uroviricota\|class_Caudoviricetes\|order_Caudovirales\|genus_mgv_g_4000221\|species_OTU-65104 |
| OTU-57202 | -2.7734 | 9.22E-03 | phylum_Uroviricota\|class_Caudoviricetes\|order_Caudovirales\|genus_mgv_g_4002154\|species_OTU-57202 |
| OTU-35031 | 2.6768 | 1.02E-02 | phylum_Uroviricota\|class_Caudoviricetes\|order_Caudovirales\|genus_mgv_g_4000834\|species_OTU-35031 |
| OTU-20918 | 4.1425 | 1.03E-02 | phylum_Uroviricota\|class_Caudoviricetes\|order_Caudovirales\|genus_Lilyvirus\|species_OTU-20918 |
| OTU-52713 | -3.2857 | 1.04E-02 | phylum_Uroviricota\|class_Caudoviricetes\|order_Caudovirales\|genus_mgv_g_4000108\|species_OTU-52713 |
| OTU-14180 | -2.5088 | 1.06E-02 | phylum_Uroviricota\|class_Caudoviricetes\|order_Caudovirales\|genus_mgv_g_4000068\|species_OTU-14180 |
| OTU-67435 | 4.7232 | 1.21E-02 | phylum_Uroviricota\|class_Caudoviricetes\|order_Caudovirales\|genus_mgv_g_4001074\|species_OTU-67435 |
| OTU-65284 | -1.8072 | 1.31E-02 | phylum_Uroviricota\|class_Caudoviricetes\|order_Caudovirales\|genus_mgv_g_4001054\|species_OTU-65284 |
| OTU-39589 | 2.9371 | 1.34E-02 | phylum_Uroviricota\|class_Caudoviricetes\|order_Caudovirales\|family_Siphoviridae\|genus_mgv_g_4000000\|species_OTU-39589 |
| OTU-57370 | -3.5683 | 1.54E-02 | phylum_Uroviricota\|class_Caudoviricetes\|order_Caudovirales\|family_Siphoviridae\|genus_mgv_g_4000007\|species_OTU-57370 |
| OTU-09469 | 3.1106 | 1.71E-02 | phylum_Uroviricota\|class_Caudoviricetes\|order_Caudovirales\|genus_mgv_g_4001693\|species_OTU-09469 |
| OTU-09943 | -2.2131 | 1.78E-02 | phylum_Uroviricota\|class_Caudoviricetes\|order_Caudovirales\|family_Siphoviridae\|genus_mgv_g_4000001\|species_OTU-09943 |
| OTU-31483 | 3.7747 | 1.83E-02 | phylum_Uroviricota\|class_Caudoviricetes\|order_Caudovirales\|genus_mgv_g_4000812\|species_OTU-31483 |
| OTU-15522 | -3.5189 | 1.85E-02 | phylum_Uroviricota\|class_Caudoviricetes\|order_Caudovirales\|genus_mgv_g_4000026\|species_OTU-15522 |
| OTU-29378 | 4.4016 | 1.86E-02 | phylum_Uroviricota\|class_Caudoviricetes\|order_Caudovirales\|genus_mgv_g_4000595\|species_OTU-29378 |
| OTU-67046 | 5.2655 | 1.91E-02 | phylum_Uroviricota\|class_Caudoviricetes\|order_Caudovirales\|genus_mgv_g_4000092\|species_OTU-67046 |
| OTU-72814 | 3.4326 | 1.98E-02 | phylum_Uroviricota\|class_Caudoviricetes\|order_Caudovirales\|family_Myoviridae\|genus_mgv_g_4000062\|species_OTU-72814 |
| OTU-38589 | 2.8904 | 2.10E-02 | phylum_Uroviricota\|class_Caudoviricetes\|order_Caudovirales\|family_Siphoviridae\|genus_mgv_g_4000093\|species_OTU-38589 |
| OTU-28545 | -1.5583 | 2.21E-02 | phylum_Uroviricota\|class_Caudoviricetes\|order_Caudovirales\|genus_mgv_g_4000221\|species_OTU-28545 |
| OTU-06802 | 2.4578 | 2.22E-02 | phylum_Uroviricota\|class_Caudoviricetes\|order_Caudovirales\|family_Siphoviridae\|genus_mgv_g_4000371\|species_OTU-06802 |
| OTU-53052 | 2.8596 | 2.23E-02 | phylum_Uroviricota\|class_Caudoviricetes\|order_Caudovirales\|genus_mgv_g_4001406\|species_OTU-53052 |
| OTU-47793 | -4.2606 | 2.30E-02 | phylum_Uroviricota\|class_Caudoviricetes\|order_Caudovirales\|genus_mgv_g_4000016\|species_OTU-47793 |
| OTU-58669 | 4.8676 | 2.31E-02 | phylum_Uroviricota\|class_Caudoviricetes\|order_Caudovirales\|family_Siphoviridae\|genus_mgv_g_4000115\|species_OTU-58669 |
| OTU-62859 | 5.1685 | 2.49E-02 | phylum_Uroviricota\|class_Caudoviricetes\|order_Caudovirales\|genus_mgv_g_4000040\|species_OTU-62859 |
| OTU-38768 | 2.9769 | 2.71E-02 | phylum_Uroviricota\|class_Caudoviricetes\|order_Caudovirales\|family_Siphoviridae\|genus_mgv_g_4000338\|species_OTU-38768 |
| OTU-27152 | 2.1428 | 2.79E-02 | phylum_Uroviricota\|class_Caudoviricetes\|order_Caudovirales\|family_Myoviridae\|genus_mgv_g_4000160\|species_OTU-27152 |
| OTU-48794 | 3.1441 | 2.81E-02 | phylum_Uroviricota\|class_Caudoviricetes\|order_Caudovirales\|genus_mgv_g_4000899\|species_OTU-48794 |
| OTU-69866 | 2.1769 | 2.92E-02 | phylum_Uroviricota\|class_Caudoviricetes\|order_Caudovirales\|genus_mgv_g_4000068\|species_OTU-69866 |
| OTU-52176 | -5.0304 | 2.92E-02 | phylum_Uroviricota\|class_Caudoviricetes\|order_Caudovirales\|family_Siphoviridae\|genus_mgv_g_4000162\|species_OTU-52176 |
| OTU-46073 | -3.5491 | 2.94E-02 | phylum_Uroviricota\|class_Caudoviricetes\|order_Caudovirales\|genus_mgv_g_4000016\|species_OTU-46073 |
| OTU-44248 | 1.5454 | 3.02E-02 | phylum_Uroviricota\|class_Caudoviricetes\|order_Caudovirales\|family_Siphoviridae\|genus_mgv_g_4000673\|species_OTU-44248 |
| OTU-68465 | 4.1630 | 3.14E-02 | phylum_Uroviricota\|class_Caudoviricetes\|order_Caudovirales\|family_Myoviridae\|genus_mgv_g_4000327\|species_OTU-68465 |
| OTU-60484 | -2.5933 | 3.18E-02 | phylum_Uroviricota\|class_Caudoviricetes\|order_Caudovirales\|genus_mgv_g_4000092\|species_OTU-60484 |
| OTU-16275 | -2.6928 | 3.32E-02 | phylum_Uroviricota\|class_Caudoviricetes\|order_Caudovirales\|genus_mgv_g_4000519\|species_OTU-16275 |
| OTU-04193 | -2.2497 | 3.34E-02 | phylum_Uroviricota\|class_Caudoviricetes\|order_Caudovirales\|genus_mgv_g_4000841\|species_OTU-04193 |
| OTU-62748 | 4.0057 | 3.34E-02 | phylum_Uroviricota\|class_Caudoviricetes\|order_Caudovirales\|genus_mgv_g_4000223\|species_OTU-62748 |
| OTU-34798 | 4.3203 | 3.37E-02 | phylum_Uroviricota\|class_Caudoviricetes\|order_Caudovirales\|genus_mgv_g_4000040\|species_OTU-34798 |
| OTU-66940 | -2.2019 | 3.39E-02 | phylum_Uroviricota\|class_Caudoviricetes\|order_Caudovirales\|genus_mgv_g_4000016\|species_OTU-66940 |
| OTU-03342 | 2.2301 | 3.44E-02 | phylum_Uroviricota\|class_Caudoviricetes\|order_Caudovirales\|family_Myoviridae\|genus_mgv_g_4000160\|species_OTU-03342 |
| OTU-33561 | -2.2508 | 3.48E-02 | phylum_Uroviricota\|class_Caudoviricetes\|order_Caudovirales\|genus_mgv_g_4000189\|species_OTU-33561 |
| OTU-17864 | 3.3836 | 3.49E-02 | phylum_Uroviricota\|class_Caudoviricetes\|order_Caudovirales\|family_Siphoviridae\|genus_mgv_g_4000319\|species_OTU-17864 |
| OTU-53240 | -3.1896 | 3.49E-02 | phylum_Uroviricota\|class_Caudoviricetes\|order_Caudovirales\|genus_mgv_g_4000016\|species_OTU-53240 |
| OTU-44420 | -2.5508 | 3.52E-02 | phylum_Uroviricota\|class_Caudoviricetes\|order_Caudovirales\|genus_mgv_g_4000228\|species_OTU-44420 |
| OTU-33879 | 1.4942 | 3.52E-02 | phylum_Uroviricota\|class_Caudoviricetes\|order_Caudovirales\|family_Siphoviridae\|genus_mgv_g_4000096\|species_OTU-33879 |
| OTU-31703 | -2.2501 | 3.60E-02 | phylum_Uroviricota\|class_Caudoviricetes\|order_Caudovirales\|genus_mgv_g_4000005\|species_OTU-31703 |
| OTU-45126 | -2.0632 | 3.60E-02 | phylum_Uroviricota\|class_Caudoviricetes\|order_Caudovirales\|family_Siphoviridae\|genus_mgv_g_4001016\|species_OTU-45126 |
| OTU-02151 | 2.0301 | 3.62E-02 | phylum_Uroviricota\|class_Caudoviricetes\|order_Caudovirales\|genus_mgv_g_4000022\|species_OTU-02151 |
| OTU-18282 | -1.9855 | 3.71E-02 | phylum_Uroviricota\|class_Caudoviricetes\|order_Caudovirales\|family_Myoviridae\|genus_mgv_g_4000292\|species_OTU-18282 |
| OTU-26739 | -2.0869 | 3.74E-02 | phylum_Uroviricota\|class_Caudoviricetes\|order_Caudovirales\|genus_mgv_g_4000085\|species_OTU-26739 |
| OTU-63879 | -2.7034 | 3.78E-02 | phylum_Uroviricota\|class_Caudoviricetes\|order_Caudovirales\|genus_mgv_g_4001066\|species_OTU-63879 |
| OTU-66750 | -2.7141 | 3.81E-02 | phylum_Uroviricota\|class_Caudoviricetes\|order_Caudovirales\|genus_mgv_g_4000030\|species_OTU-66750 |
| OTU-19133 | 3.7214 | 3.88E-02 | phylum_Uroviricota\|class_Caudoviricetes\|order_Caudovirales\|family_Myoviridae\|genus_mgv_g_4000073\|species_OTU-19133 |
| OTU-24080 | 2.8459 | 3.90E-02 | phylum_Uroviricota\|class_Caudoviricetes\|order_Caudovirales\|genus_mgv_g_4000065\|species_OTU-24080 |
| OTU-05838 | -2.2997 | 3.93E-02 | phylum_Uroviricota\|class_Caudoviricetes\|order_Caudovirales\|family_Siphoviridae\|genus_mgv_g_4000196\|species_OTU-05838 |
| OTU-74662 | 3.4723 | 3.94E-02 | phylum_Uroviricota\|class_Caudoviricetes\|order_Caudovirales\|family_Siphoviridae\|genus_mgv_g_4000319\|species_OTU-74662 |
| OTU-66232 | 3.1470 | 3.95E-02 | phylum_Uroviricota\|class_Caudoviricetes\|order_Caudovirales\|family_Siphoviridae\|genus_mgv_g_4000561\|species_OTU-66232 |
| OTU-73684 | -2.5295 | 4.01E-02 | phylum_Uroviricota\|class_Caudoviricetes\|order_Caudovirales\|genus_mgv_g_4000696\|species_OTU-73684 |
| OTU-29135 | -2.2369 | 4.29E-02 | phylum_Uroviricota\|class_Caudoviricetes\|order_Caudovirales\|genus_mgv_g_4000005\|species_OTU-29135 |
| OTU-64243 | 1.9386 | 4.36E-02 | phylum_Uroviricota\|class_Caudoviricetes\|order_Caudovirales\|family_Myoviridae\|genus_Svunavirus\|species_OTU-64243 |
| OTU-50636 | -1.6552 | 4.42E-02 | phylum_Uroviricota\|class_Caudoviricetes\|order_Caudovirales\|genus_mgv_g_4001017\|species_OTU-50636 |
| OTU-23434 | -2.1064 | 4.48E-02 | phylum_Uroviricota\|class_Caudoviricetes\|order_Caudovirales\|genus_mgv_g_4000227\|species_OTU-23434 |
| OTU-26665 | -2.2832 | 4.53E-02 | phylum_Uroviricota\|class_Caudoviricetes\|order_Caudovirales\|genus_mgv_g_4000323\|species_OTU-26665 |
| OTU-25220 | -2.9109 | 4.56E-02 | phylum_Uroviricota\|class_Caudoviricetes\|order_Caudovirales\|genus_mgv_g_4000022\|species_OTU-25220 |
| OTU-15753 | -2.4697 | 4.58E-02 | phylum_Uroviricota\|class_Caudoviricetes\|order_Caudovirales\|genus_mgv_g_4000016\|species_OTU-15753 |
| OTU-32682 | 1.6542 | 4.77E-02 | phylum_Uroviricota\|class_Caudoviricetes\|order_Caudovirales\|genus_mgv_g_4000085\|species_OTU-32682 |
| OTU-38709 | -2.7003 | 4.80E-02 | phylum_Uroviricota\|class_Caudoviricetes\|order_Caudovirales\|genus_mgv_g_4000016\|species_OTU-38709 |
| OTU-54621 | 1.7889 | 4.81E-02 | genus_mgv_g_4000215\|species_OTU-54621 |
| OTU-46021 | -1.7501 | 4.84E-02 | phylum_Uroviricota\|class_Caudoviricetes\|order_Caudovirales\|family_Siphoviridae\|genus_mgv_g_4000009\|species_OTU-46021 |
| OTU-16669 | -1.9146 | 4.94E-02 | phylum_Uroviricota\|class_Caudoviricetes\|order_Caudovirales\|family_Siphoviridae\|genus_mgv_g_4000196\|species_OTU-16669 |
| OTU-35327 | -1.8506 | 4.97E-02 | phylum_Uroviricota\|class_Caudoviricetes\|order_Caudovirales\|genus_mgv_g_4000095\|species_OTU-35327 |
| OTU-12414 | -2.4425 | 5.08E-02 | phylum_Uroviricota\|class_Caudoviricetes\|order_Caudovirales\|genus_mgv_g_4000672\|species_OTU-12414 |
| OTU-11729 | -1.8006 | 5.12E-02 | phylum_Uroviricota\|class_Caudoviricetes\|order_Caudovirales\|family_Siphoviridae\|genus_mgv_g_4000419\|species_OTU-11729 |
| OTU-19344 | -2.9376 | 5.13E-02 | phylum_Uroviricota\|class_Caudoviricetes\|order_Caudovirales\|family_Siphoviridae\|genus_mgv_g_4000136\|species_OTU-19344 |
| OTU-02532 | -3.1017 | 5.14E-02 | phylum_Uroviricota\|class_Caudoviricetes\|order_Caudovirales\|genus_mgv_g_4000026\|species_OTU-02532 |
| OTU-18906 | -1.7349 | 5.29E-02 | phylum_Uroviricota\|class_Caudoviricetes\|order_Caudovirales\|family_Siphoviridae\|genus_mgv_g_4001048\|species_OTU-18906 |
| OTU-65811 | -3.0507 | 5.40E-02 | phylum_Uroviricota\|class_Caudoviricetes\|order_Caudovirales\|genus_mgv_g_4000060\|species_OTU-65811 |
| OTU-60197 | -2.1520 | 5.43E-02 | phylum_Uroviricota\|class_Caudoviricetes\|order_Caudovirales\|genus_mgv_g_4000672\|species_OTU-60197 |
| OTU-47236 | -1.8264 | 5.54E-02 | phylum_Uroviricota\|class_Caudoviricetes\|order_Caudovirales\|family_Siphoviridae\|genus_mgv_g_4000001\|species_OTU-47236 |
| OTU-18637 | -1.5045 | 5.62E-02 | phylum_Uroviricota\|class_Caudoviricetes\|order_Caudovirales\|genus_Lilyvirus\|species_OTU-18637 |
| OTU-49265 | -2.6855 | 5.63E-02 | phylum_Uroviricota\|class_Caudoviricetes\|order_Caudovirales\|genus_mgv_g_4000005\|species_OTU-49265 |
| OTU-33930 | 2.7188 | 5.80E-02 | phylum_Uroviricota\|class_Caudoviricetes\|order_Caudovirales\|genus_mgv_g_4000016\|species_OTU-33930 |
| OTU-46698 | -1.8092 | 5.80E-02 | phylum_Uroviricota\|class_Caudoviricetes\|order_Caudovirales\|family_Siphoviridae\|genus_mgv_g_4000001\|species_OTU-46698 |
| OTU-30083 | 1.0555 | 5.92E-02 | phylum_Uroviricota\|class_Caudoviricetes\|order_Caudovirales\|family_Siphoviridae\|genus_mgv_g_4000001\|species_OTU-30083 |
| OTU-17654 | 4.8911 | 6.08E-02 | phylum_Uroviricota\|class_Caudoviricetes\|order_Caudovirales\|genus_mgv_g_4000005\|species_OTU-17654 |
| OTU-45373 | -2.1976 | 6.19E-02 | phylum_Uroviricota\|class_Caudoviricetes\|order_Caudovirales\|family_Siphoviridae\|genus_mgv_g_4000076\|species_OTU-45373 |
| OTU-35882 | -1.2686 | 6.42E-02 | phylum_Phixviricota\|class_Malgrandaviricetes\|order_Petitvirales\|family_Microviridae\|genus_mgv_g_4000019\|species_OTU-35882 |
| OTU-06403 | 3.0116 | 6.62E-02 | phylum_Uroviricota\|class_Caudoviricetes\|order_Caudovirales\|family_Siphoviridae\|genus_mgv_g_4000419\|species_OTU-06403 |
| OTU-05170 | 3.3541 | 6.74E-02 | phylum_Uroviricota\|class_Caudoviricetes\|order_Caudovirales\|genus_mgv_g_4000022\|species_OTU-05170 |
| OTU-50196 | -2.4000 | 6.77E-02 | phylum_Uroviricota\|class_Caudoviricetes\|order_Caudovirales\|genus_mgv_g_4000108\|species_OTU-50196 |
| OTU-51226 | -2.1916 | 6.90E-02 | phylum_Uroviricota\|class_Caudoviricetes\|order_Caudovirales\|genus_mgv_g_4000005\|species_OTU-51226 |
| OTU-45276 | 2.2449 | 6.90E-02 | phylum_Uroviricota\|class_Caudoviricetes\|order_Caudovirales\|family_Siphoviridae\|genus_mgv_g_4000673\|species_OTU-45276 |
| OTU-03879 | -1.9277 | 7.04E-02 | phylum_Uroviricota\|class_Caudoviricetes\|order_Caudovirales\|family_Siphoviridae\|genus_mgv_g_4000147\|species_OTU-03879 |
| OTU-44956 | -1.5355 | 7.43E-02 | phylum_Uroviricota\|class_Caudoviricetes\|order_Caudovirales\|genus_mgv_g_4000069\|species_OTU-44956 |
| OTU-17994 | 1.4560 | 7.65E-02 | phylum_Uroviricota\|class_Caudoviricetes\|order_Caudovirales\|genus_mgv_g_4001256\|species_OTU-17994 |
| OTU-21190 | 2.0181 | 7.85E-02 | phylum_Uroviricota\|class_Caudoviricetes\|order_Caudovirales\|family_Siphoviridae\|genus_mgv_g_4000180\|species_OTU-21190 |
| OTU-33741 | -1.5933 | 8.03E-02 | phylum_Uroviricota\|class_Caudoviricetes\|order_Caudovirales\|genus_mgv_g_4001973\|species_OTU-33741 |
| OTU-46395 | 2.9000 | 8.27E-02 | phylum_Uroviricota\|class_Caudoviricetes\|order_Caudovirales\|family_Siphoviridae\|genus_mgv_g_4000096\|species_OTU-46395 |
| OTU-55622 | 1.0956 | 8.44E-02 | phylum_Hofneiviricota\|class_Faserviricetes\|order_Tubulavirales\|family_Inoviridae\|genus_mgv_g_4000202\|species_OTU-55622 |
| OTU-58336 | 1.6002 | 8.44E-02 | phylum_Uroviricota\|class_Caudoviricetes\|order_Caudovirales\|family_Siphoviridae\|genus_mgv_g_4000689\|species_OTU-58336 |
| OTU-45424 | 1.9839 | 8.46E-02 | phylum_Uroviricota\|class_Caudoviricetes\|order_Caudovirales\|family_Myoviridae\|genus_mgv_g_4000251\|species_OTU-45424 |
| OTU-34487 | -2.8543 | 8.72E-02 | phylum_Uroviricota\|class_Caudoviricetes\|order_Caudovirales\|family_Siphoviridae\|genus_mgv_g_4000007\|species_OTU-34487 |
| OTU-43708 | -1.9315 | 9.12E-02 | phylum_Uroviricota\|class_Caudoviricetes\|order_Caudovirales\|genus_mgv_g_4000005\|species_OTU-43708 |
| OTU-69103 | 2.6340 | 9.15E-02 | phylum_Uroviricota\|class_Caudoviricetes\|order_Caudovirales\|family_Siphoviridae\|genus_mgv_g_4000018\|species_OTU-69103 |
| OTU-35848 | 3.0977 | 9.33E-02 | phylum_Uroviricota\|class_Caudoviricetes\|order_Caudovirales\|family_Myoviridae\|genus_mgv_g_4000073\|species_OTU-35848 |
| OTU-61816 | 2.3732 | 9.92E-02 | phylum_Uroviricota\|class_Caudoviricetes\|order_Caudovirales\|genus_mgv_g_4000005\|species_OTU-61816 |
